# Malaria hotspots and climate change trends in the hyper-endemic malaria settings of Mizoram along the India–Bangladesh borders

**DOI:** 10.1038/s41598-023-31632-6

**Published:** 2023-03-20

**Authors:** Pachuau Lalmalsawma, K. Balasubramani, Meenu Mariya James, Lalfakzuala Pautu, Kumar Arun Prasad, Devojit Kumar Sarma, Praveen Balabaskaran Nina

**Affiliations:** 1 Integrated Disease Surveillance Programme, Health and Family Welfare Department, Aizawl, Mizoram India; 2 Department of Geography, School of Earth Sciences, Central University of Tamil Nadu, Thiruvarur, Tamil Nadu India; 3 Department of Epidemiology and Public Health, Central University of Tamil Nadu, Thiruvarur, Tamil Nadu India; 4 Department of Life Sciences, Pachhunga University College, Mizoram University, Aizawl, Mizoram India; 5 ICMR- National Institute for Research in Environmental Health, Bhopal, Madhya Pradesh India; 6 Department of Public Health and Community Medicine, Central University of Kerala, Kasaragod, Kerala India

**Keywords:** Climate-change impacts, Malaria, Epidemiology

## Abstract

India has made tremendous progress in reducing malaria mortality and morbidity in the last decade. Mizoram State in North-East India is one of the few malaria-endemic regions where malaria transmission has continued to remain high. As Mizoram shares international borders with Bangladesh and Myanmar, malaria control in this region is critical for malaria elimination efforts in all the three countries. For identifying hotspots for targeted intervention, malaria data from 385 public health sub-centers across Mizoram were analyzed in the Geographic Information System. Almost all the sub-centers reporting high Annual Parasite Index (> 10) are located in Mizoram’s districts that border Bangladesh. Getis-Ord G_i_* statistic shows most of the sub-centers located along the Bangladesh border in the Lawngtlai and Lunglei districts to be the malaria hotspots. The hotspots also extended into the Mamit and Siaha districts, especially along the borders of Lawngtlai and Lunglei. Analysis of terrain, climatic, and land use/land cover datasets obtained from the Global Modelling and Assimilation Office and satellite images show Mizoram’s western part (Lawngtlai, Lunglei, and Mamit districts) to experience similar topographic and climatic conditions as the bordering Rangamati district in the Chittagong division of Bangladesh. Climatic trends in this region from 1981 to 2021, estimated by the Mann–Kendall test and Sen's slope estimates, show an increasing trend in minimum temperature, relative humidity, rainfall, and the associated shift of climatic pattern (temperate to tropical monsoon) could facilitate malaria transmission. The quasi-Poisson regression model estimates a strong association (*p* < 0.001) between total malaria cases, temperature range, and elevation. The Kruskal–Wallis H test shows a statistically significant association between malaria cases and forest classes (*p* < 0.001). A regional coordination and strategic plan are required to eliminate malaria from this hyper-endemic malaria region of North-East India.

## Introduction

Globally, in 2020, malaria cases and deaths were estimated to be 241 million and 627,000 respectively^1^. Compared to 2019, this represents a 5.8% increase in cases and an 11% increase in deaths; ~ 2/3rd of the additional deaths (47,000) in 2020 have been attributed to the disruption in malaria prevention and control efforts by the COVID-19 pandemic^2^. Even though the pandemic was predicted to increase malaria incidence and deaths in 2020, the phenomenal progress made since 2000 (27% and 51% decrease in case incidence and mortality, respectively) has started plateauing around 2017^2^.

In India, in the last decade, malaria cases and deaths have declined significantly; 1018 deaths in 2010 have steeply decreased to 93 in 2020^1^. Among the 11 most malaria-endemic countries, India is the only one to report a reduction in cases in 2020, compared to 2019^1^. Despite the steep decrease in malaria incidence across India, there are a few endemic pockets where the decline in malaria incidence has been much lower than the national average and continues to pose a stiff challenge to India’s malaria elimination efforts. The state of Mizoram in North-East (NE) India is one such hyper-endemic malaria region, where malaria remains a major public health challenge.

The Indo-Myanmar region where Mizoram is located in one of the top 10 biodiversity hotspots in the world^3^. A majority of Mizoram’s population belongs to the Kuki Chin group, with an ancestral link to the Tibeto-Burma branch of the Indo-Mongloid race^4^. Mizoram is dominated by tribals (~ 94%)^5^, receives heavy rainfall (~ 2000 mm annually), and an estimated 89% of Mizoram (18,748 sq. km.) is under forest cover^6^ with Jhum cultivation practiced in 63% of the total crop area^7^. Malaria in Mizoram is predominantly (> 90%) transmitted by *Plasmodium falciparum*^8^. *Anopheles minimus* and *An. dirus* have been incriminated as malaria vectors in Mizoram^9^. While in the rest of India, Artemisinin combination therapy (ACT) is Artesunate plus Sulfadoxine-Pyrimethamine (AS-SP), in NE India (including Mizoram), SP resistance has led to the replacement of SP with the lumefantrine in the ACT (artemether-lumefantrine)^10^. Mizoram shares historical ties and porous international borders with the bordering districts of Myanmar and Bangladesh^4,11^. Widespread artemisinin resistance has been reported in Myanmar, and a recent study has indicated the emergence of artemisinin resistance independent of *kelch13* mutations in Bangladesh^12^. Mizoram is considered to be one of the major routes for the entry of drug-resistant parasites from Southeast Asia (SEA) to NE India^8,13^. India, Bangladesh, and Myanmar have all committed to malaria eradication by 2030; the porous borders shared by Mizoram with these two countries could allow the movement of parasites and vectors in either-directions jeopardizing the malaria elimination efforts of all three countries.

In many parts of the world, malaria is seasonal and is influenced by short term changes in the meteorological variables—temperature, rainfall and humidity^14^. Temperature is a key factor that influences both the parasite and vector development; vector’s life cycle from hatching of egg to progression of various developmental stages is influenced by temperature^15,16^. Malaria parasite’s sporogonic cycle that determines the vectorial capacity and disease incidence is affected by temperature^17^ , and so is the vector’s gonotrophic cycle that defines the frequency of mosquito-human interactions^16^. Rainfall (provides vector breeding sites) and suboptimal relative humidity (reduces mosquito’s activity and life span) are other key environmental factors affecting malaria incidence^16,18–21^. The elevation is also associated with the intensity of disease transmission; low temperatures in elevated regions make it hard for the vectors to survive, resulting in lower vector densities and reduced/no disease transmission^22–26^. Given the hyperendemicity of malaria in Mizoram, it would be pertinent to study the role of environmental factors affecting malaria transmission in this region.

Geographic Information System (GIS) helps in identifying the geographical distribution of cases, spatio-temporal links of the disease, spatial clusters of the disease, and the population at risk^27^. Worldwide, GIS has been used as an effective tool to combat infectious diseases, including malaria^28–31^. As Mizoram shares porous international borders with Myanmar and Bangladesh, it is important to understand the spatio-temporal distribution of malaria cases for devising regional intervention strategies. Here, using GIS, we have mapped the clusters of malaria cases at the sub-health centre level across Mizoram from 2015 to 21, and have identified the hotspots. The environmental variables (temperature, humidity, rainfall, and land use/land cover) were also mapped to study their association with malaria. Furthermore, climate change over the last 4 decades and how it might influence malaria transmission in this region are discussed.

## Methods

### Data source and mapping

A malaria case is a person in whom the presence of malaria parasite in the blood has been confirmed by diagnostic tests. Malaria surveillance is carried out by the public health sub-center, which is the smallest unit, and the data will be reported to the district health headquarters on a daily basis. The data is collected mainly by passive surveillance, however, at the village level, active surveillance using Rapid Diagnostic Test (RDT) is also carried out in fever cases by Accredited Social Health Activist (ASHA) workers, who report to public health sub-centers. The public health sub-center level malaria datasets from 2015 to 2021 were collected from the Directorate of Health Services, Mizoram. The datasets represent 385 sub-centers (after data cleaning) across nine districts (Lawngtlai, Lunglei, Kolasib, Mamit, Aizawl East, Aizawl West, Siaha, Champhai, and Serchhip) of Mizoram. The datasets were compiled and prepared as a geodatabase comprising the key variables: total malaria cases, percentage of *Plasmodium falciparum, Plasmodium vivax* and Annual Parasite Index (API). The datasets used in the study is provided in Supplementary Table 1. API is calculated based on the total number of positive slides for the parasite in a year × 1000/Total population of a particular area. The population size under the surveillance of each health sub-center is surveyed and recorded by the respective centers. As the sub-center datasets are maintained by the district health headquarters, collection of the sub-center datasets for the entire state is tedious. Some of the districts provided only the district-level datasets to the Directorate of Health Services (Mizoram), and data were missing for Lawngtlai (2015–2018), Siaha (2018), and Aizawl East (2018) districts. Hence, in addition to the sub-center malaria datasets, district-wise datasets were also prepared for mapping the spatio-temporal distributions. The total malaria cases at district-level were normalized using the projected population of 2020, based on the population estimates of Wang et.al.^32^. All available API data of each health sub-center during 2015–2021 was used to estimate the average API.

To characterize the regional settings of Mizoram, climatic variables, digital terrain model, and land use/land cover (LU/LC) datasets of the Global Modelling and Assimilation Office (GMAO)^33^ and NASA satellite missions were retrieved from NASA Power^34^, LP DAAC^35^, and USGS Earth Explorer^36^ tools, respectively. The long-term gridded climatic datasets representing the annual average values covering the border districts of Lunglei and Lawngtlai (Mizoram), and Rangamati (Bangladesh) were extracted from 1981 to 2021 (41 years) and used for long-term trend analysis of temperature (minimum, maximum and range), relative humidity, and rainfall. In addition, present and future climate classification maps were used to understand the shift in regional climatic pattern^37^. The LU/LC map of Mizoram and adjacent regions for the year 2022 was prepared using Landsat 8 satellite images. The details of satellite images used in the study are given in Supplementary Table S2. Supervised classification logic was adopted for preparing eight LU/LC classes by selecting training samples using ERDAS Imagine software. The parametric rule-based maximum likelihood classification method was used to extract the LU/LC information^38^. Accuracy assessment was carried out with 160 stratified random sample locations (a minimum of 10 per LU/LC class) by comparing high-resolution datasets (Google Earth images) and published LU/LC maps (International Geospatial-Biosphere Programme—https://lpdaac.usgs.gov/products/mcd12q1v006/ and Bhuvan- https://bhuvan-app1.nrsc.gov.in/thematic/thematic/index.php). The overall classification accuracy is 85.63%, and the Kappa coefficient is 0.8450, which ensures an acceptable level of accuracy.

To extract environmental variables for statistical analysis, a one km buffer was used around the public health sub-centers. The values of elevation, climate, and LU/LC parameters within the buffer zone were extracted using the Zonal Statistics tool in ArcGIS 10.4 software (https://desktop.arcgis.com), and the mean value for each parameter was obtained. All the layers were prepared as spatial layers and mapped in the ArcGIS 10.4 software using dot, choropleth, and isopleth mapping techniques.

### Statistical analysis

Out of the eight LU/LC classes identified in the study area, three classes (water, wetland, and cropland) were not considered for the analysis as they were not present in the buffer zone of any of the public health sub-center. The rest of the LU/LC classes are grouped into one land use class (built-up/Jhum), and two land cover classes (mixed forest/shrub, and dense forest). The Kruskal–Wallis H test was used to examine if the total number of malaria cases was different for (a) built-up/Jhum (n = 14), (b) mixed forest/shrub (n = 110), and (c) dense forest (n = 261) classes.

To estimate the association between the total malaria cases in all districts of Mizoram and the independent variables (elevation, minimum temperature, maximum temperature, temperature range, rainfall, and relative humidity), a generalized linear regression model was used. The dependent variable (total malaria cases) is highly skewed (Skewness = 9.1 and Kurtosis = 116.7) (Supplementary Table S3) and multicollinearity issue was observed among the independent variables in the generalized linear regression analysis (Supplementary File S4). In addition, pair-wise correlation/association of all variables was determined using a correlation matrix (Supplementary Table S5), which showed a high correlation between the temperature minimum, temperature range, and temperature maximum. A high correlation was also observed between elevation, relative humidity, and rainfall. Therefore, temperature minimum and rainfall were not considered for the multiple regression analysis using a quasi-Poisson regression model. The statistical significance was determined using the adjusted incidence rate ratio (aIRR), which is obtained by exponentiating the coefficients of a quasi-Poisson regression model. The statistical analysis was performed using STATA 17 (StataCorp, USA) and ArcGIS Pro 3.0 (ESRI, USA) software.

### Optimized hotspots analysis

To understand the sub-center level hotspots of malaria in Mizoram, the Optimized Hotspot Analysis tool in ArcGIS Pro 3.0 software was used. We used all available year-wise Annual Parasite Index (API) of the 385 public health sub-centers spread across the state (Supplementary File S6). The hotspots were identified based on the Getis-Ord G_i_* statistic, using z-scores (standard deviations) and *p*-values (probability), of each sub-centers within the context of the neighboring sub-centers^39^. Extreme z-scores (very high or positive and very low or negative) with significant *p*-values, populating in the tails of the normal distribution curve are termed hot and cold spots, respectively. In our study, we have used multiple significant levels (90%, 95%, and 99% confidence intervals) for better understanding.

The Getis-Ord G_i_* statistics is given as,

1 $$G_{i}^{*} = \frac{{\sum\nolimits_{j}^{n} {w_{i,j} \,x_{j} - \overline{X} \sum\nolimits_{j}^{n} {w_{i,j} } } }}{{S\sqrt[{}]{{\frac{{\left[ {n\sum\nolimits_{j = 1}^{n} {w_{{_{i,j} }}^{2} } - \left( {\sum\nolimits_{j = 1}^{n} {w_{i,j} } } \right)^{2} } \right]}}{n - 1}}}}}$$

2 $$\overline{X} = \frac{{\sum\nolimits_{j = 1}^{n} {x_{j} } }}{n},\,\,S = \sqrt {\frac{{\sum\nolimits_{j = 1}^{n} {x_{{_{j} }}^{2} } }}{n}} - (\overline{X} )^{2}$$

where *x*_*j*_ is the API for the particular sub-center *j* in the selected year, *w*_*i,j*_ is the spatial weight between neighboring sub-centers *i* and *j*, and* n* is the total number of sub-centers involved in our analysis^40^.

We could decide whether to accept the null hypothesis or not using the *p*-value from the significance test obtained for the G_i_* statistic^41^.

3 $$Z_{G} = \frac{(G - E(G))}{{\sqrt {V(G)} }}$$

Here, E(G) represents the expected G, and V(G) is the variance of G, which can be calculated as

4 $$E(G) = \frac{{\sum\limits_{i = 1}^{n} {\sum\limits_{j = 1}^{n} {W_{ij} (d)} } }}{n(n - 1)},\forall j \ne i$$

5 $$V(G) = E(G^{2} ) - E(G)^{2}$$

When the absolute value of the Z-score is large and the *p*-value is less than 0.05, the null hypothesis can be rejected i.e., the sub-centers representing API exhibit statistically significant clustering.

### Trend analysis

The MAKESENS template developed for time series analysis by the Finnish Meteorological Institute was used to estimate the trends in the climatic (temperature, rainfall, humidity) variables over 41 years. The MAKESENS estimates the presence of a trend (either increasing or decreasing) using the nonparametric Mann–Kendall test, while its magnitude (true slope of the linear trend) is estimated by the Sens’s slope estimate^42,43^.

### Ethical approval

This study is a secondary data analysis and does not involve experiments with humans/or the use of human tissue samples, therefore, human ethical approval is not applicable.

## Results

### Characteristics of study site

**Figure 1:**
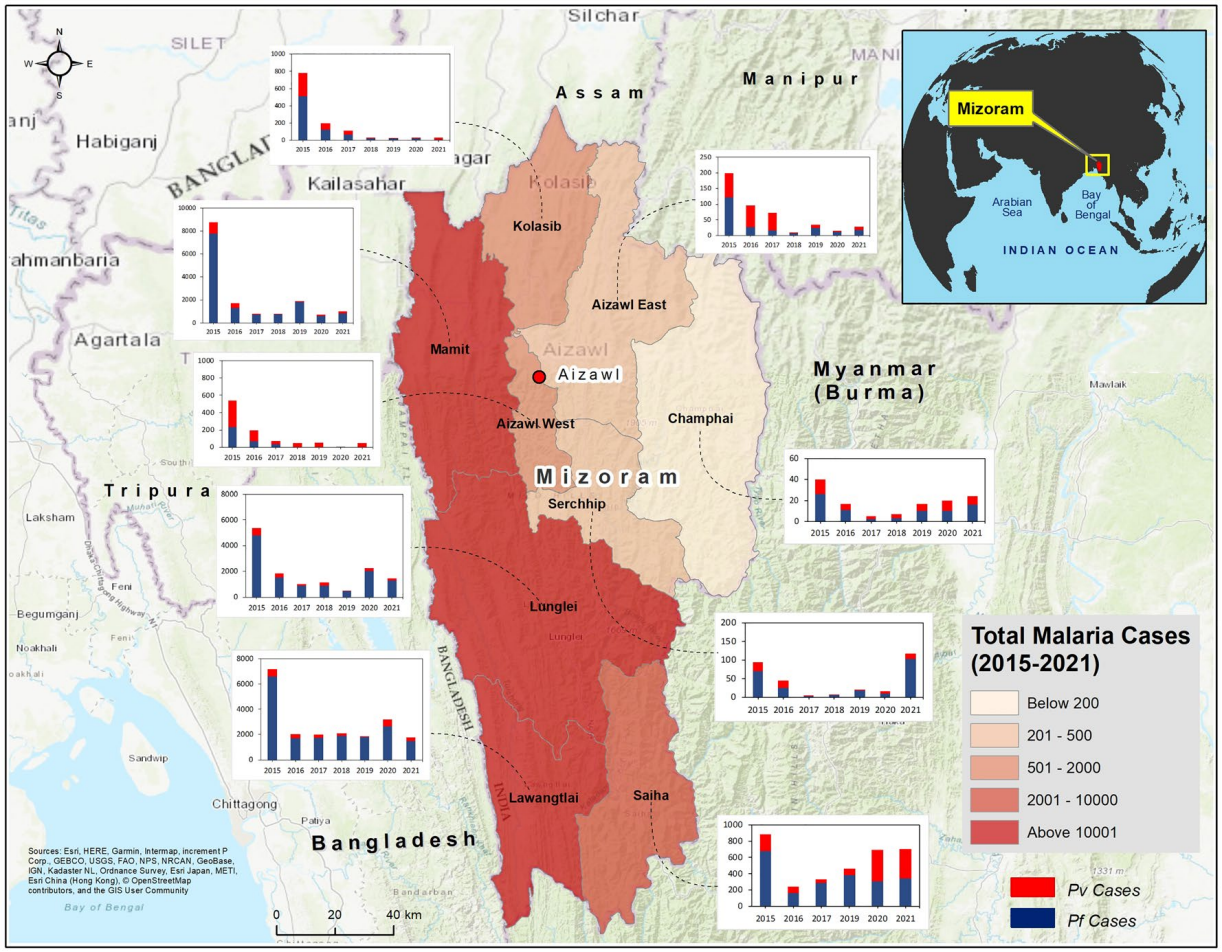
Location map of Mizoram showing the total number of Malaria cases from 2015 to 2021. The color ramp shows the distribution of caseloads with darker for higher and light for lower. Stacked bar charts for each districts represents the trend of *Pf* and *Pv* from 2015 to 2021. The map was created using licensed version of ArcGIS 10.4 software by Esri. The background map represents World Topographic Map. For more information about the base map services, visit http://goto.arcgisonline.com/maps/World_Topo_Map.

Mizoram, located in NE India, shares the majority of its western borders with the Chittagong Hill Tracts of Bangladesh, and its eastern and southern borders with the Chin Hills of Myanmar (Fig. 1). Among the eight major districts (Mamit, Kolasib, Aizawl, Champhai, Serchhip, Lunglei, Lawngtlai, and Siaha) in Mizoram, Lunglei (4536 sq. km) and Aizawl (3576 sq. km) are the largest. Aizawl, the capital, has the highest (78.6%) urban population, and Mamit has the highest (82.7%) rural population. Across Mizoram, the maximum and minimum mean temperature ranges from 33.9 to 35.7 °C and 11.3 to 14.0 °C respectively. The annual average rainfall is highest in Mamit (1807 mm), and lowest in Champhai (925 mm). Champhai and Lawngtlai have the highest and lowest mean elevation, respectively. The state’s LU/LC is dominated by evergreen forests and shrublands. At least 90% of the land area is covered by different categories forests in each of the districts^44^. Detailed district-wise demographic and geographic characteristics of Mizoram are given in Supplementary Table S7, and the LU/LC distribution (in sq. km) is given in Supplementary Table S8.

### Descriptive epidemiology

During the study period (2015–2021), 55,778 malaria cases were reported across the nine districts of Mizoram. Figure 1 shows the cumulative malaria cases from 2015 to 21, and the increase in caseloads from the Bangladesh border (lower elevation) to the Myanmar border (higher elevation). The highest number of cases were reported from Lawngtlai (20,074), followed by Mamit (15,631), and Lunglei (13,580); these districts share international borders with Bangladesh. A total of 47,287 (84.78%) *Pf* cases and 8,491 (15.2%) *Pv* cases were recorded. Both *Pf* and *Pv* cases were highest in Lawngtlai district; 37.78% (17,865 cases) and 26.02% (2209 cases), respectively. However, the proportions of *Pv* cases were highest in Siaha and Aizawl districts (> 50%). The average API throughout the study period across districts was 8.40. The highest average API was reported in 2015 (27.14), and the lowest in 2018 (4.33). From 23,849 cases in 2015, a steady decrease in malaria prevalence was observed till 2018 (4221). In the last three years (2019–21), cases have gradually increased; 4870 cases (2019), 6951 cases (2020), and 5166 cases (2021) (Supplementary Table S1).

**Figure 2:**
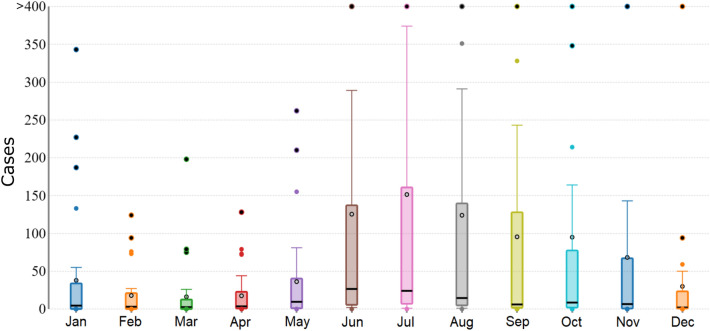
Monthly distribution of total malaria cases in Lunglei, Serchhip, and Mamit districts of Mizoram (2015–2021).

The monthly distribution of total malaria cases in the Lunglei, Serchhip, and Mamit districts is shown in Fig. 2. The cases peak from June to September, which is the monsoon season in Mizoram^45^. Supplementary Fig. 9 shows the distribution of the API across the sub-centers, along with the total malaria cases reported in each districts, from 2015 to 2021. Throughout the study period, the sub-centers located in the western districts of Mizoram, especially Mamit, Lunglei, and Lawngtlai, which share international borders with Bangladesh, exhibited high API. As we move towards the eastern parts of Mizoram, a significant reduction in malaria cases was observed. Till 2019, API was reported to be below 1 in the sub-centers along the Myanmar border, but a surge in API can be seen in 2020 and 2021, where four sub-centers of Champhai reported an increase in API (1.1–10) and one center each in Lunglei and Serchhip reported API above 100 in 2021. Overall, the average API of Mizoram showed a decreasing trend, from 27.22 in 2015 to 5.55 in 2021. The number of sub-centers with API > 10 reduced from 2015 to 2018; however, the numbers have started to rise from 2019 to 2021. Except for two, all the sub-centers reporting high API (> 10) are located in the districts that border Bangladesh. Even though sub-center-wise data is not available for Lawngtlai (2015–2018), Siaha (2018), and Aizawl East (2018), the district-level aggregated data indicate the cases were endemic along the Bangladesh border throughout the study period.

**Figure 3:**
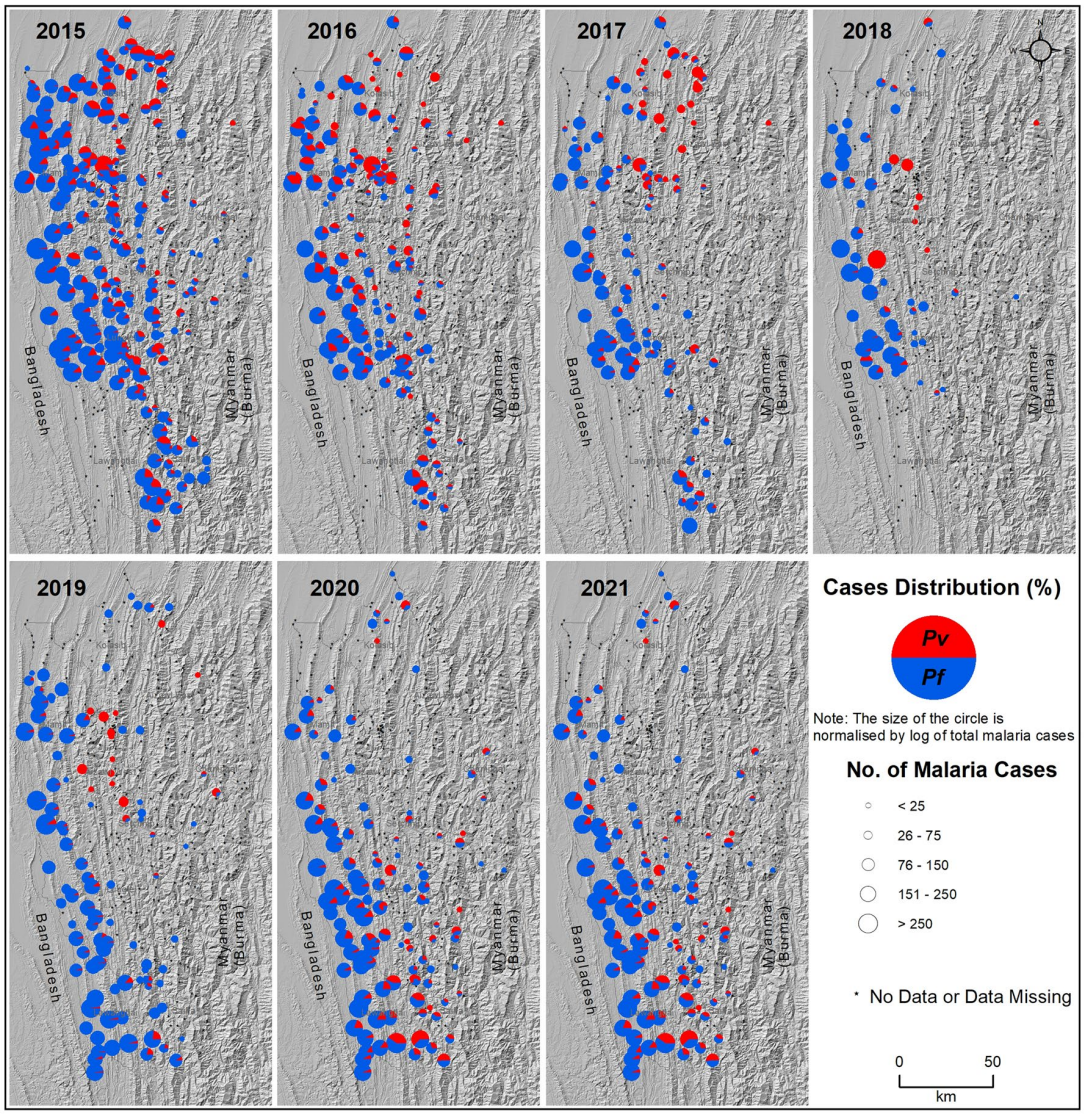
Distribution of *Plasmodium falciparum* (blue) and *Plasmodium vivax* (red) cases across the sub-centers of Mizoram from 2015 to 2021. The (large) size of the circles denotes the (high) malaria cases. Note that sub-center-wise data is not available for some of the years in Lawngtlai (2015–2018), Siaha (2018), and Aizawl East (2018) districts. The background topographic layer was prepared using NASA SRTM (Shuttle Radar Topography Mission) Global 30 arc second DEM data accessed from https://doi.org/10.5067/MEaSUREs/SRTM/SRTMGL30.002. The map layout was created using licensed version of ArcGIS 10.4 software by Esri. For more information about Esri software, please visit www.esri.com.

The distribution of *Pf* and *Pv* cases across the sub-centers of Mizoram from 2015 to 2021, with the terrain as a base map, is shown in Fig. 3. Throughout the study period, a high number of cases were reported in the sub-centers in the plain regions and the least number of cases in the high terrains. In 2015, 87.11% of the malaria cases were *Pf*, and it consistently remained the majority over the study period (77.50% in 2016, 85.93% in 2017, 86.93% in 2018, 92.36% in 2019, 80.85% in 2020, and 78.59% in 2021). The total *Pv* cases were 12.89% in 2015, and it increased to 22.50% in 2016; then, there was a gradual decline until 2019 (14.07% in 2017, 13.31% in 2018, and 7.64% in 2019). A steep increase was observed again in 2020 and 2021, with 19.15% and 21.41% of the total malaria cases being *Pv*, respectively. Most of the sub-centers with *Pv* cases are located in the urban districts (northern parts of Mizoram) of Aizawl East, Aizawl West, and Kolasib, whereas the sub-centers in the rural districts (mid and southern parts of Mizoram) of Mamit, Lunglei, and Lawngtlai, predominantly reported *Pf* infections. In recent years (2020–21), the proportion of *Pv* cases has increased considerably in the high-burden southern rural districts of Lawngtlai and Siaha.

### Spatial clustering analysis

**Figure 4:**
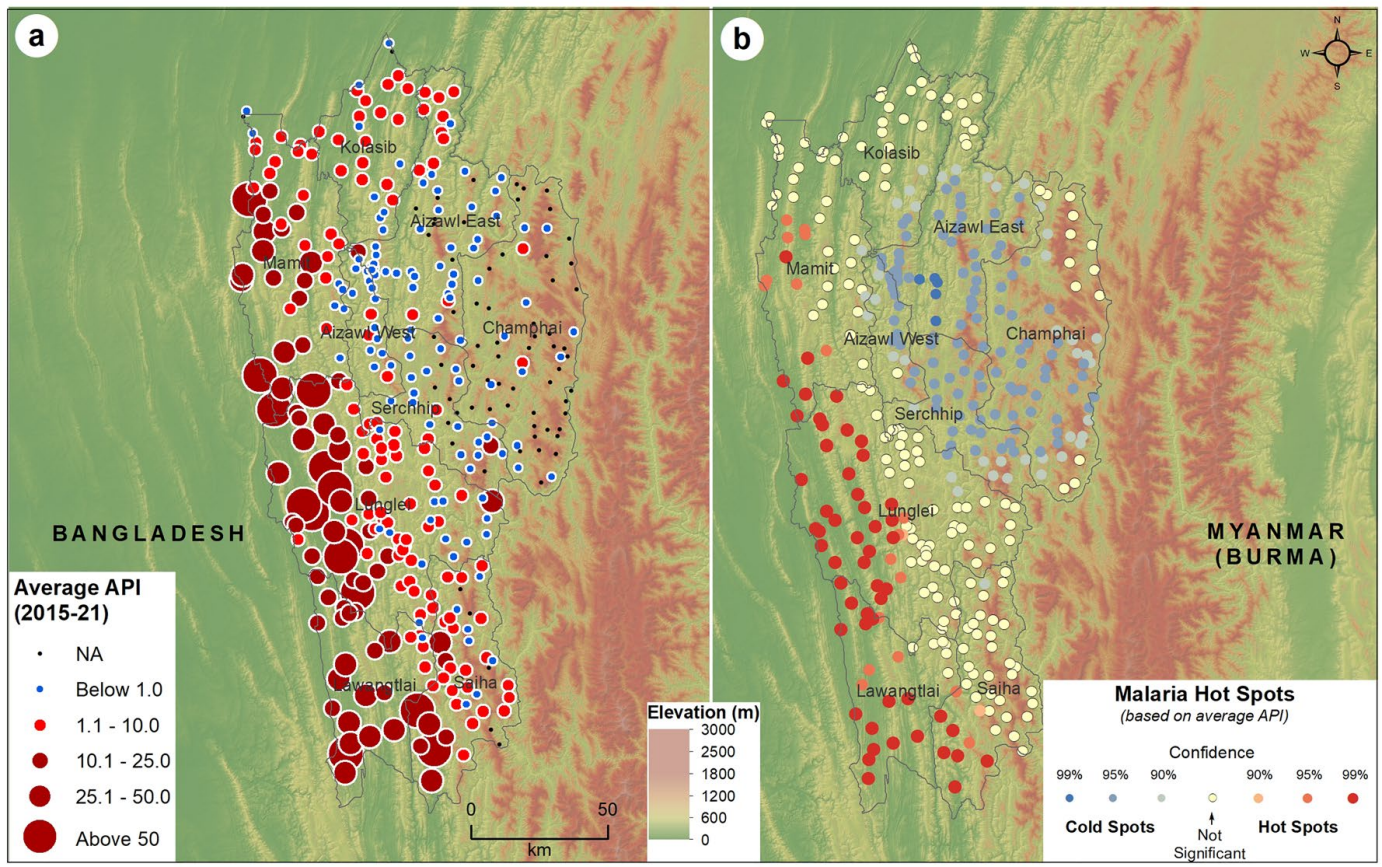
Distribution of (**a**) average Annual Parasite Index (2015–2021) and (**b**) spatial clusters of malaria cases in Mizoram based on average Annual Parasite Index. The large size circle with dark red color (**a**) shows the higher values (> 10) of API. The red and blue shaded dots represent (**b**) the hot and cold spots, respectively of malaria cases in Mizoram with different statistical significance. The yellow-colored dots are not statistically significant for forming hot or cold spots. The background shades represent the elevation above mean sea level extracted using NASA SRTM (Shuttle Radar Topography Mission) Global 30 arc second DEM data available at https://doi.org/10.5067/MEaSUREs/SRTM/SRTMGL30.002. The map layout was created using licensed version of ArcGIS 10.4 software by Esri. For more information about Esri software, please visit www.esri.com.

The average API reported in the sub-centers of Mizoram from 2015 to 2021, with a base map of elevation, is shown in Fig. 4a. The sub-centers located in the western districts of Mizoram, especially Mamit, Lawngtlai, and Lunglei, reported a high API during the study period, where the elevation was relatively low (plains). The API gradually decreased from the Bangladesh-Mizoram border to the Mizoram-Myanmar border, as the elevation increased. The majority of the sub-centers with an API > 10.1 are located in the plain regions of Mizoram, while an API of 1.1–10.1 was observed in the transition zones, and towards the highly elevated areas, the majority of the sub-centers reported an API below 1.0. The malaria hotspots identified through the location and API of sub-centers in Mizoram are shown in Fig. 4b. Most of the sub-centers located along the Bangladesh border in the Lawngtlai and Lunglei districts are malaria hotspots. The hotspots also extended into the Mamit and Siaha districts, especially along the borders of Lawngtlai and Lunglei. Most sub-centers in Aizawl East, Aizawl West, Champhai, and Serchhip are malaria coldspots with an average API of less than one. The terrain differences determine the distribution of hot and cold spots; cold spots are in the higher elevated regions, while hotspots are at lower elevation.

The evolution of the malaria hotspots from 2015 to 2021 in Mizoram is shown in Supplementary Figure S10. The sub-centers in the Western border of Mizoram, primarily in Mamit, Lunglei, and Lawngtlai, are consistently the hotspots for malaria over the study period. In 2020 and 2021, the hotspots extended eastwards, with the emergence of malaria hotspots in the sub-centers of Siaha. A similar pattern was observed with the evolution of *Pf* hotspots in Mizoram (Supplementary Figure S11), as the majority of the total cases are *Pf*.

**Figure 5:**
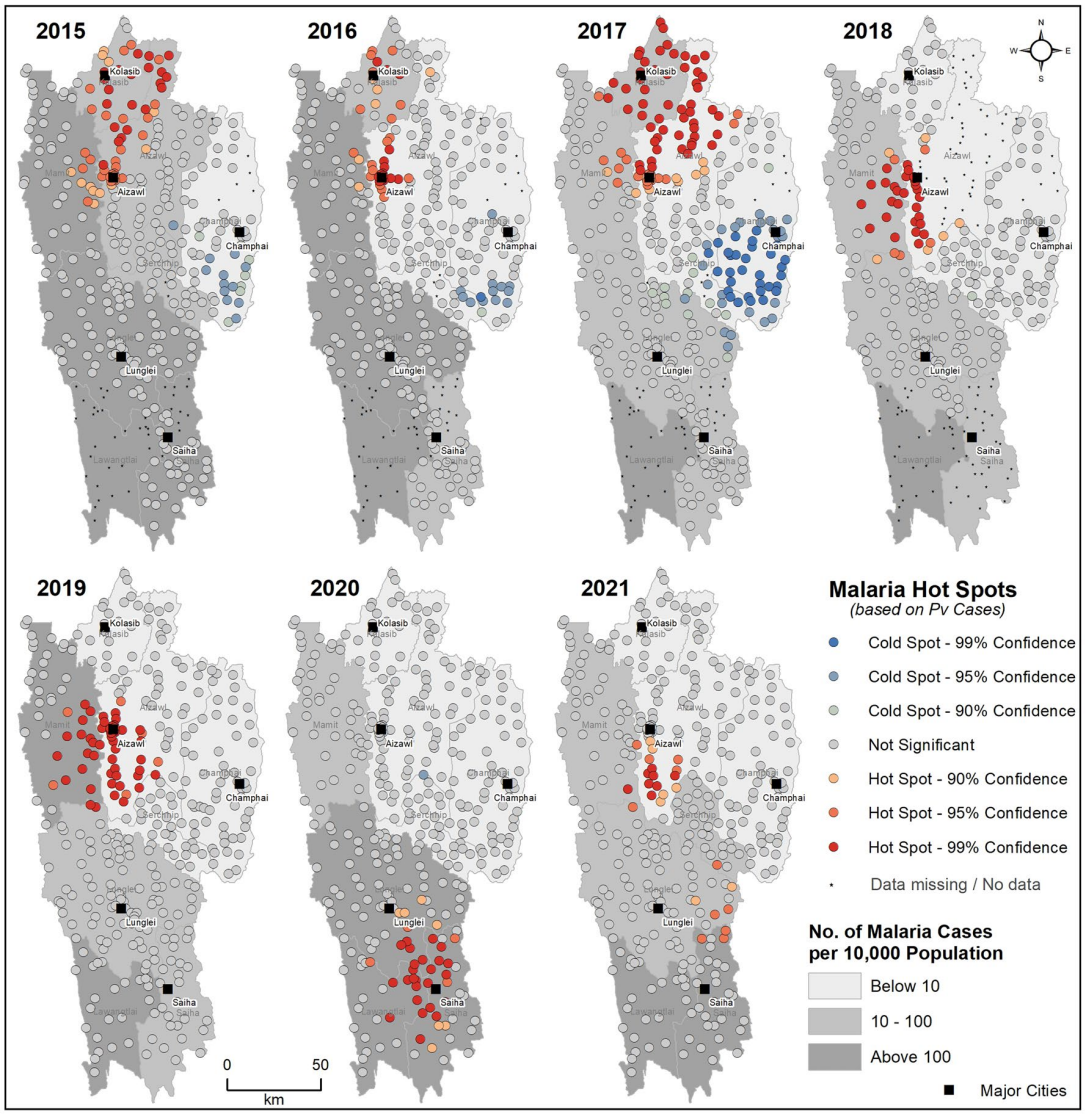
Space–time trends of *Pv* malaria hotspots from 2015 to 2021 in Mizoram. The map was created using licensed version of ArcGIS 10.4 software by Esri. For more information about Esri software, please visit www.esri.com.

*Pv* hotspots were primarily concentrated in the sub-centers of Aizawl and Kolasib cities from 2015 to 2017. From 2018 to 2019, a shift in the *Pv* hotspots towards the south and western parts of Aizawl was observed. The majority of the sub-centers surrounding the city of Siaha emerged as hotspots for *Pv* in 2020 (Fig. 5).

### Association with environmental variables

**Figure 6:**
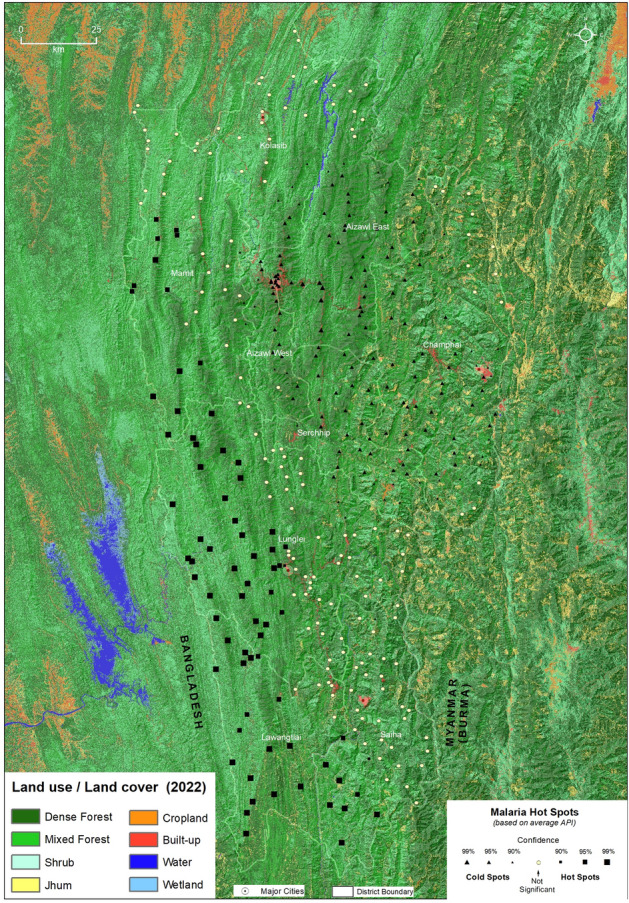
Distribution of the land use/land cover (LU/LC) in Mizoram and adjoining regions in 2022. The LU/LC classes were extracted from Landsat 8 images extracted from https://earthexplorer.usgs.gov and are draped over digital elevation model (DEM) of NASA SRTM (Shuttle Radar Topography Mission) Global 30 arc second DEM data available at https://doi.org/10.5067/MEaSUREs/SRTM/SRTMGL30.002 for better visualization. The map was created using licensed version of ArcGIS 10.4 software by Esri. For more information about Esri software, please visit www.esri.com.

The spatial distribution of LU/LC of Mizoram and the bordering areas is shown in Fig. 6. The shrubland is the dominant LU/LC type along the Western border of Mizoram, especially in Mamit, Lunglei, and Lawngtlai. The LU/LC was dominated by dense forests towards the Eastern border. A Jhum type of cultivation is dominant in the Eastern hilly terrains, especially in the Champhai district.

**Figure 7:**
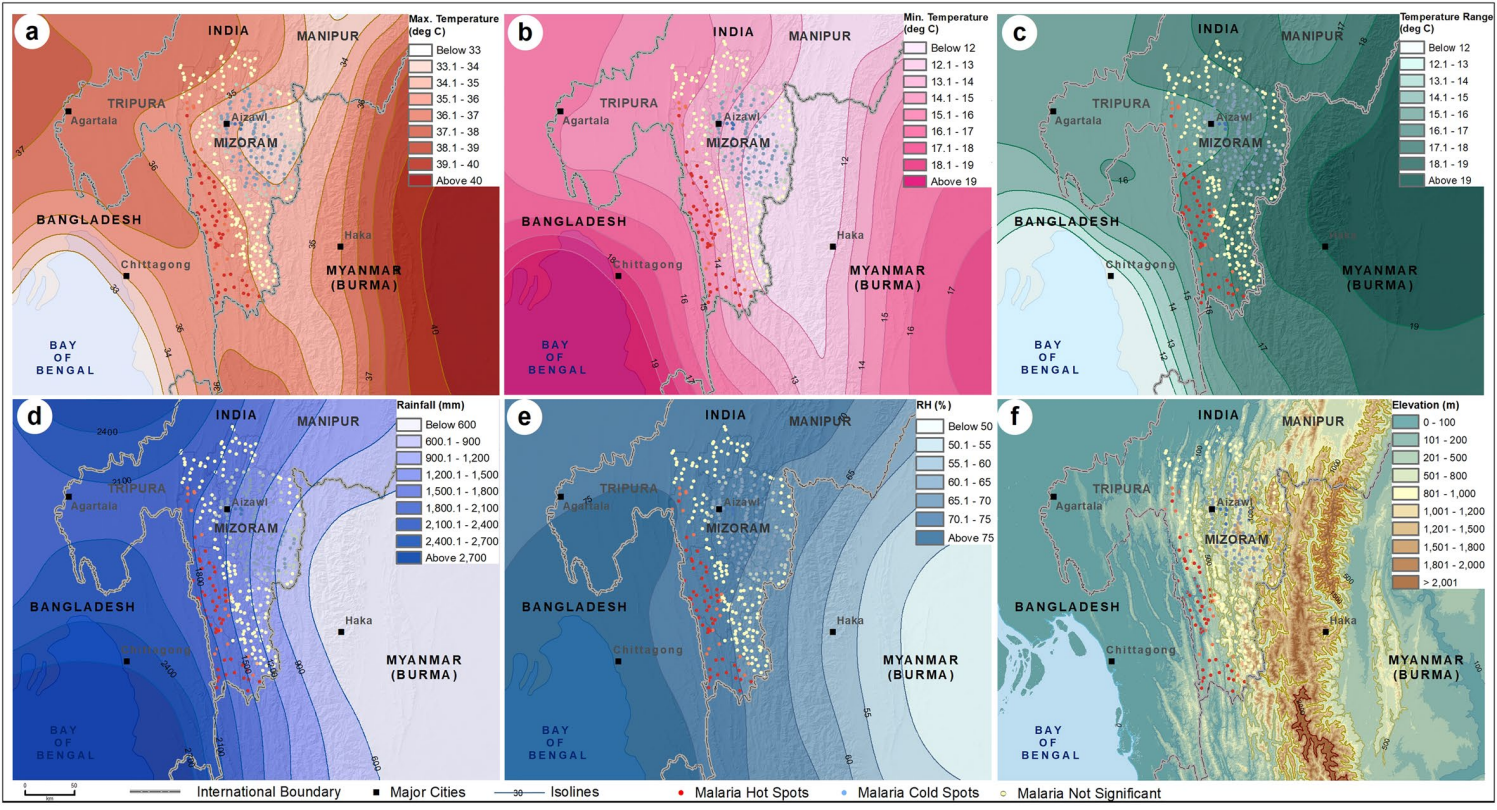
Regional distribution of average (**a**) Maximum Temperature, (**b**) Minimum Temperature, (**c**) Temperature Range, (**d**) Rainfall, (**e**) Relative Humidity, and (**f**) elevation. The legend of each figure is given separately. The darker shades of color ramp in figures represent relatively higher values. The climatic datasets were extracted from https://power.larc.nasa.gov/data-access-viewer/ and terrain data was extracted from https://doi.org/10.5067/MEaSUREs/SRTM/SRTMGL30.002. The map layout was created using licensed version of ArcGIS 10.4 software by Esri. For more information about Esri software, please visit www.esri.com.

The spatial distribution of maximum temperature, minimum temperature, temperature range, rainfall, and relative humidity from 1981 to 2021 are shown in Fig. 7. Analysis of the annual meteorological data from 1981 to 2021 shows the western districts of Mizoram experience similar climatic conditions as the bordering districts located in the Chittagong division of Bangladesh. The terrain settings of Mizoram, along with Eastern and Western border regions, are also presented in Fig. 7.

The spatial clusters of malaria are overlaid in the maps of environmental variables for a better understanding of malaria and its environmental settings (Figs. 6 and 7). The LU/LC distribution shows the malaria hotspots of Mizoram are primarily located in the shrublands that border the mixed or dense forests. Such a pattern of LU/LC is also observed in the Chittagong Hill Tract of Bangladesh, with a transition of deciduous forests to shrublands to croplands. Analysis showed a statistically significant difference in the number of malaria cases between the mean ranks of at least one pair of groups, χ^2^ (2) = 19.652, *p* < 0.001. The pairwise comparison matrix estimated by the Kruskal–Wallis H test also shows a significant positive association (*p* < 0.0001) of forest categories (dense, mixed, and shrub forests) and malaria cases (Table 1 and Supplementary Table 12).

**Table 1:** Pairwise comparisons (Kruskal–Wallis H test) of land use and malaria cases.

Pairwise	Test statistic	Std. error	Std. test statistic	Sig.	Adj. Sig.^a^
Built-up/Jhum—Dense Forest	− 45.554	30.475	− 1.495	0.135	0.405
Built-up/Jhum—Mixed Forest/Shrub	− 95.538	31.522	− 3.031	0.002	0.007*
Dense forest—Mixed Forest /Shrub	49.984	12.628	3.958	0.000	0.000*

^a^Significance values have been adjusted by the Bonferroni correction for multiple tests. *Significant at *p* < 0.05.

The spatial overlay of malaria clusters and climatic parameters shows the maximum temperature experienced in the malaria hotspot regions of Mizoram is 35–36 °C (Figure 7a), the minimum temperature is 13–15 °C (Fig. 7b), and the average diurnal range is 15–17 °C (Fig. 7c). Malaria hotspots were primarily clustered in Lawngtlai, Lunglei, Mamit, and Siaha districts which are with relatively higher temperatures (maximum is above 35 °C and the minimum is 13–14 °C), whereas coldspots were observed in the elevated districts (Champhai, Aizawl, and Serchhip) with relatively lower temperatures (maximum is less than 34 °C and the minimum is less than 13 °C). A similar pattern is observed with the average rainfall and relative humidity (Fig. 7d and e). The average annual rainfall and relative humidity in this hotspot region are 1500–2100 mm and 70–75%, respectively. All these climatic parameters are identical in the bordering regions of Bangladesh and the neighboring Indian state of Tripura. The annual average temperature (maximum and minimum), rainfall, and relative humidity displayed a decreasing pattern from the Chittagong Hill Tract of Bangladesh towards the Myanmar border of Mizoram (Fig. 7a,b,d, and e). All these LU/LC and climatic parameters are highly interrelated with the regional elevation (Fig. 7f). All the hotspots fall within 500 m (above MSL) elevation, and the coldspots are located predominantly above 1000 m (above MSL).

The multivariate association between the total malaria cases and environmental variables—temperature (min, max, and range), elevation, relative humidity, and LU/LC estimated by the quasi-Poisson regression model is presented in Table 2. A statistically significant association was observed between the total malaria cases and the environmental variables (temperature range, elevation, dense forest, and mixed forest/scrub). Total malaria cases significantly increased with a decrease in the temperature range (aIRR 0.220; 95%CI 0.099–0.490) and elevation (aIRR 0.997; 95%CI 0.995–0.999). The dense forest (aIRR 3.843; 95%CI 1.881–7.852) and mixed forest/shrub (aIRR 5.395; 95%CI 2.652–10.975) have a higher rate of malaria cases.

**Table 2:** Association between total malaria cases and the environmental variables.

Total Malaria Cases	uIRR	95% CI		*p*-value	aIRR	95% CI		*p*-value
Temp. Max (°C)	3.838	3.020	4.879	0.000	1.117	0.486	2.568	0.794
Temp. range (°C)	0.212	0.150	0.298	0.000	0.220	0.099	0.490	0.000
Relative humidity (%)	1.711	1.419	2.062	0.000	0.901	0.663	1.225	0.507
Elevation (m)	0.996	0.995	0.997	0.000	0.997	0.995	0.999	0.000
Dense forest	0.370	0.191	0.719	0.003	3.843	1.881	7.852	0.000
Mixed forest/shrub	3.171	1.637	6.146	0.001	5.395	2.652	10.975	0.000

uIRR: unadjusted Incidence Rate Ratios, aIRR: adjusted Incidence Rate Ratios.

### Climatic trend analysis

**Figure 8:**
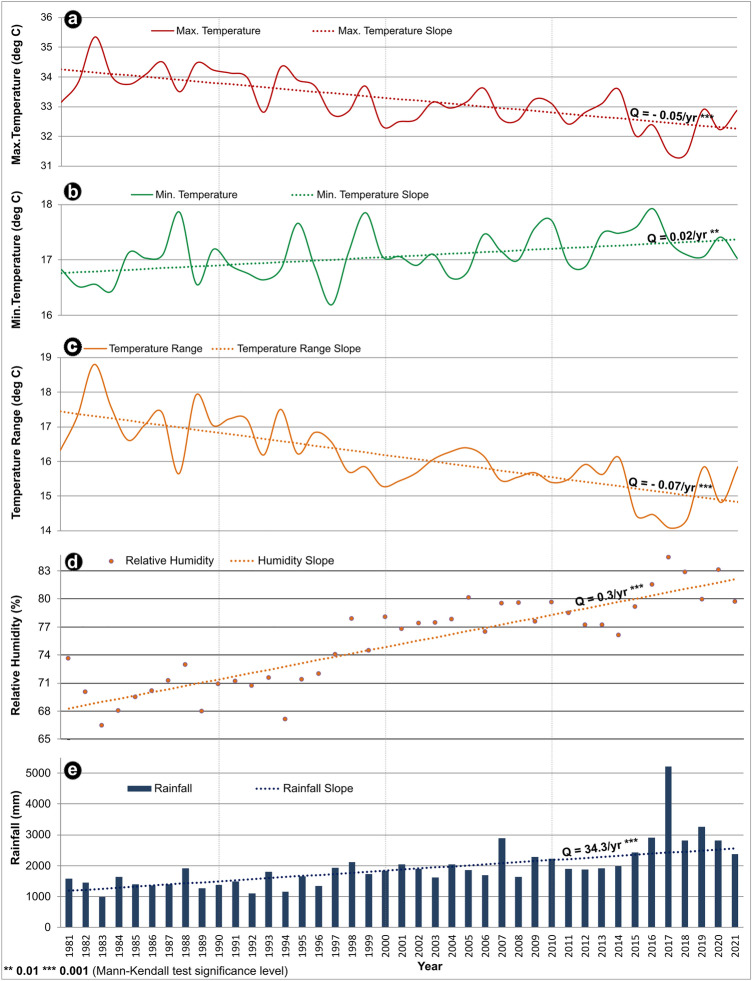
Long-term trends in (**a**) Maximum temperature; (**b**) Minimum temperature; (**c**) Temperature range; (**d**) Relative humidity; (**e**) Rainfall in the Western region of Mizoram. The dotted lines in the graphs show Sen’s slope, and the rate of change per year (Q) with the statistical significance (based on the Z-value of the Mann–Kendall test) is appended above the line.

The climatic trend in the malaria hotspot (Western border) and cold-spot regions (Eastern border) of Mizoram from 1981 to 2021 are shown in Fig. 8, Supplementary Figure S13, and Supplementary Table S14. A decreasing trend in the maximum temperature and diurnal temperature range (Q: 0.05 °C/year and − 0.07 °C/year, at *P* < 0.001, respectively) is observed in the Western region. The minimum temperature increased at a rate of 0.015 °C/year (*P* < 0.01). Also, the relative humidity (Q = 0.3%/year; *P* < 0.001) and rainfall (Q = 34.28 mm; *P* < 0.001) of the region showed an increasing trend over the past four decades. Till 2006, the annual average rainfall recorded was below 2000 mm per year, and a significant increasing trend was observed in the past decade, with the maximum rainfall recorded in 2017 (5204 mm).

In the Eastern Border of Mizoram, the climatic trend (1981–2021) follows a similar pattern as the western part. The maximum (Q: 0.03 °C/year; *P* < 0.05) and minimum temperature, and the diurnal temperature range show a decreasing trend, while the average rainfall (Q: 19.8 °C/year; *P* < 0.001) and relative humidity (Q: 0.38 °C/year; *P* < 0.05) follow an increasing trend.

**Figure 9:**
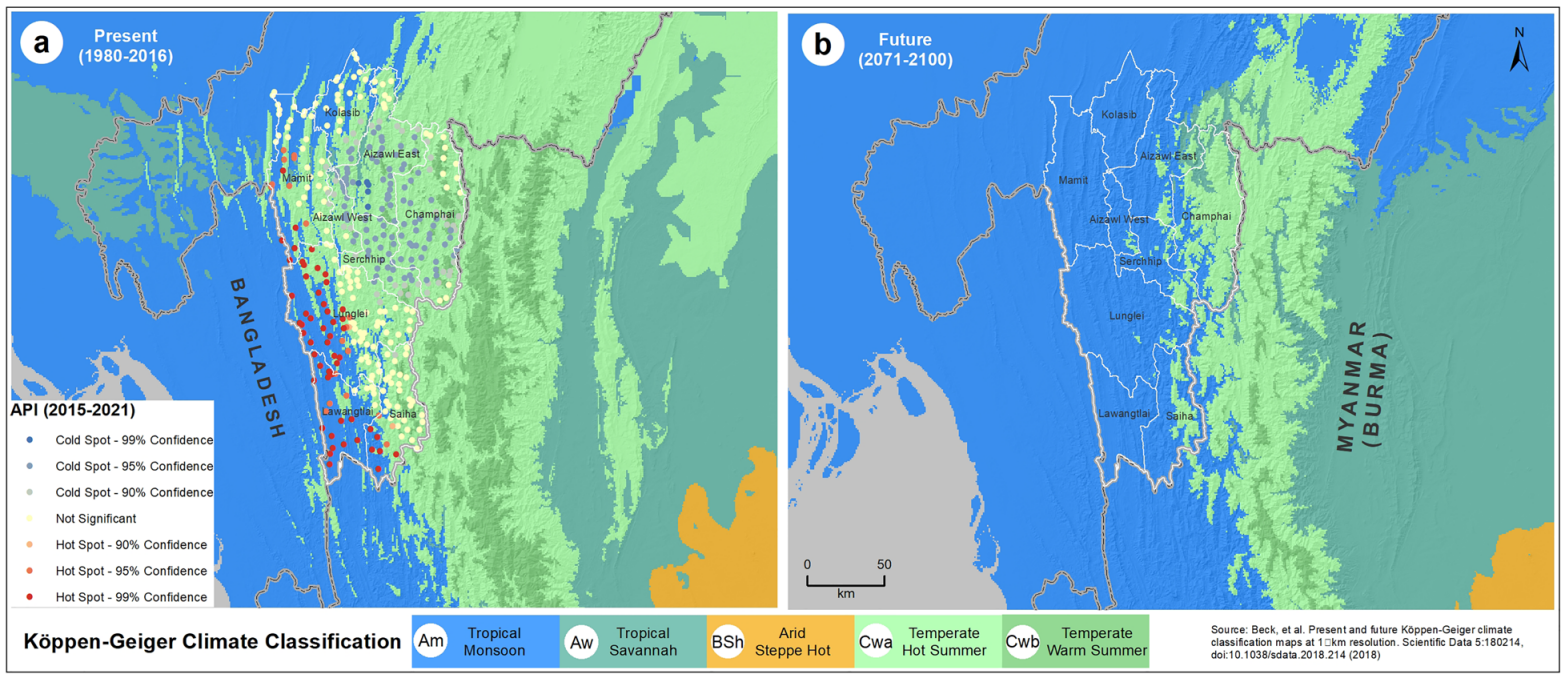
Climate types of Mizoram and adjoining regions using the Köppen-Geiger climate classification; (**a**) the present (1980–2016) and (**b**) future scenario (2071–2100) corresponding to RCP8.5 climate scenario. The map layout was created using licensed version of ArcGIS 10.4

Using the Köppen-Geiger climate classification maps^37^, the malaria hotspots are compared with the present (1980–2016) climatic types (Fig. 9a). The overlay analysis shows the malaria hotspots to fall predominately under the Tropical Monsoon (Am) type climate, which is characterized by seasonal rainfall and driest months. The future climatic map (Fig. 9b) was derived based on an ensemble of climate projections from the 32 CMIP5 models (2071–2100) corresponding to the Representative Concentration Pathway (RCP) 8.5 climate scenario. It shows the climatic types of eastern parts of Mizoram will shift from the present temperate (hot summer) type to the tropical monsoon type, which is also evidenced by the climatic trend analysis (Supplementary Figure S13).

## Discussion

In the last decade, India has seen a steep decline in malaria morbidity and mortality^46^. In most parts of the country, malaria prevalence and incidence have been consistently declining^46^. However, Mizoram is one of the few malaria-endemic pockets where the decrease in malaria prevalence has been relatively low and has even increased in the last two years, despite the COVID-19 pandemic. As per National Framework for Malaria Elimination (NFME), Mizoram falls under category 3 (intensified control phase); these are States/Union Territories having API > 1^47^. Malaria control in Mizoram is critical for India’s malaria elimination efforts, as its western and eastern districts share porous international borders with Bangladesh and Myanmar, countries that are endemic to malaria.

Mamit, Lunglei, and Lawngtlai, the western districts of Mizoram, share international borders with Chittangong Hill Tract (CHT) of Bangladesh; the CHT comprises Rangamati, Bandarban and Khagrachhari districts. Of the 64 districts in Bangladesh, malaria is endemic in 13, and 19 million people are at risk^48^. In Bangladesh, from 2008 (84,690 cases) to 2020 (6,130 cases), malaria incidence has steeply declined by ~ 93%^49^. The remaining malaria cases are from 13 of the 64 districts, and of these 13 districts, 3 (Rangamati, Bandarban and Khagrachhari) in the Chittagong Hill Tracts (CHT) contribute to ~ 90% of the malaria cases^50^. GIS analysis of public health sub-centre-wise malaria data of Mizoram from 2015 to 21 shows malaria is highly endemic in the western districts, and as we move towards the east, the malaria cases decreases steeply. It is best illustrated by the steep decrease in malaria cases as we move from Mamit district (> 10,000 cases) in the west, traversing the central districts of Kolasib/Aizawl and/or Serchhip to Champhai district (< 200) in the east. The high endemicity of malaria in the CHT and western districts of Mizoram could be attributed to the favorable meteorological parameters-temperature, humidity, and rainfall. Studies predict malaria transmission is bounded by the thermal optimum of 17 °C and 34 °C^51–53^. In addition, the relatively plain terrain of this region would also be conducive for the breeding of vectors^54^. As we move towards the East, the temperature and RH drops, and the altitude from the mean sea level increases (Fig. 7), resulting in less favorable breeding conditions for mosquitoes and malaria transmission. Similar to Aizawl (Mizoram)^21^, in CHT, the minimum temperature, RH and rainfall have shown a consistent upward trend in the last 4 decades, thus favoring mosquito breeding and malaria transmission. Multiple global climate models show an overall net increase in populations at risk and climate suitability for malaria^55^. The trend analysis shows there is a gradual shift in average maximum temperature towards less than 34 °C, minimum temperature towards 18 °C and reduction in diurnal temperature, thus favoring malaria transmission in the CHT and western districts of Mizoram. In Western Mizoram, over the last 3 decades, an estimated ~ 34 mm annual increase in rainfall is observed, and the increase is marked from 2015 onwards. If the increase in rainfall is also characterized by decreased spacing, it could lead to rapid expansion of mosquitoes^56^. Analysis of Eastern (current study) and Central Mizoram^21^ shows similar overall climatic trends as observed in the western region. Elevation in the eastern region of Mizoram appears to be a major factor limiting the spread of malaria. However, the predicted future climate change scenario indicates a tropical monsoon climate throughout Mizoram, paving the way for vector breeding and sustained malaria transmission. In the highland regions of Ethiopia and Colombia, 0.2 °C increase in temperature per decade has led to malaria transmission in these countries’ high-altitude regions^57,58^. Therefore, unless malaria transmission is stringently controlled in the CHT and western districts of Mizoram, the effect of climate change could result in a more conducive ecological niche favoring malaria transmission in the central and eastern parts of Mizoram.

After a gradual decline in malaria prevalence from 2015 to 18, Mizoram has seen an uptick in cases from 2019 onwards, especially in its western districts. This trend is worrisome, especially with escalating malaria and the recent emergence of K13 independent ART resistance in the CHT of Bangladesh^12^. Molecular screening of *Pfkelch13* in NE carried out in 2014–15 has identified non-synonymous mutations (K189T and A578S) in the Lunglei district, Mizoram^59^. ACT efficacy studies carried out in the Lunglei district from 2011 to 2013 showed no correlation between K13 propeller mutation (A578S) and treatment failures^60^. In the last decade, studies have reported delayed clearance phenotypes from parasites in Central (Chhattisgarh), South-West (Goa), and NE (Assam, Arunachal Pradesh, and Tripura) India, independent of K13 gene polymorphisms^61,62^. Decreased ART sensitivity across India and CHT and the recent increase in malaria cases in Mizoram’s international borders necessitate the need for continuous surveillance of the parasite phenotypes, especially in Mizoram’s western districts. Unlike molecular surveillance, which can be carried out in filter paper spots, assessing ART resistance through ring-stage survival assays (RSA) will be challenging as it will involve the collection and quick transport of blood samples from these arduous terrains to a malaria culture facility. Effective malaria control in the Indo-Bangladesh border districts would require coordinated efforts by both countries. Unless malaria is effectively controlled in CHT, the porous Indo-Bangladesh borders will allow the movement of parasites and vectors to Mizoram’s western districts and vice versa.

The eastern districts of Mizoram share international borders with the Chin State of Myanmar. In addition to the geographical proximity, Mizoram shares deep cultural and religious ties with Myanmar, and the people are bonded by similar customs, traditions, and beliefs^11^. In these porous borders, people from Mizoram and Myanmar continue to move in both directions, leading to the free movement of parasites in either direction, complicating malaria control. Historically, SEA has been a hotspot for the generation of acquired resistance to pyrimethamine, chloroquine, sulphadoxine, quinine, mefloquine, and most worryingly, artemisinins^8^. The first instance of drug resistance to chloroquine and SP in India has been from the NE region; these drug-resistant parasites are suspected to have entered the NE from SEA through the porous international borders^63^. In a state-wise study carried out across Mizoram from 2015 to 17, all the mutations identified in the *pfcrt* gene conferring chloroquine resistance belonged to the South-East Asian CVIET haplotype, suggesting an evolutionary link between the parasites in Mizoram and SEA^8^. In the Chin state of Myanmar that borders Mizoram, the K13 propeller mutations have been very low, and previous studies have not reported any decrease in ART sensitivity in this region^64,65^. However, in Homalin from the Sagaing region of Myanmar, 25 km from Manipur (India), ~ 47% of the parasites carried propeller mutations^64^. Mizoram shares domestic borders with Manipur State in the north^64^. Unlike the western districts bordering Bangladesh, the elevated altitude and climatic variables are less conducive to mosquito breeding and malaria transmission in the Mizoram-Myanmar border. The current absence of ART-resistant parasites does not preclude the future entry of drug-resistant parasites through Mizoram-Myanmar’s porous international borders. GIS-based analysis shows the emergence of malaria (> 10 API) in 47 sub-centers in 2021, and out of these, the highest API (> 100 API) is recorded in two sub-centers of Serchhip and Lunglei districts, bordering Myanmar. These clusters have to be monitored to assess the emergence of drug-resistant parasites.

In these high transmission malaria settings, it is also pertinent to track the status of *Pv* drug resistance. *Pv* parasites resistant to chloroquine have emerged in most *Pv* endemic countries^66^. Literature on *Pv* drug resistance is sparse in NE, and there are no reports on the status of *Pv* drug resistance in Mizoram. In India’s urban settings, *Pv* predominates^67^, and GIS analysis shows *Pv* cases to be concentrated in the urban centers of Aizawl East, Aizawl West, Kolasib and Siaha. Therapeutic efficacy studies, as assessed by response to treatment on day 28^68^, have to be carried out in Lungtian, Lungpher, Sangau, and Vawmbuk sub-centers in Lawngtlai, where *Pv* predominates.

In addition to parasite surveillance within these international borders, it is imperative to continuously assess the vector population. A district-wise mosquito survey carried out by our team at RMRC (ICMR) Dibrugarh, from late 2000 to early 2010 shows a high density of *Anopheles minimus* in Western Mizoram and South Tripura, bordering Bangladesh. As observed here, urban malaria in India is mostly transmitted by *Pv*^69^, and the reasons are not clear. It is possible specific vectors well suited for survival in urban settings might transmit *Pv* more efficiently. Also, *Pv* appears to dominate at the border between plain and elevated areas. It is possible, the ecological niche and the vectors determine the distribution of *Pv* and *Pf*. Vector epidemiology and vector-parasite relationship can change spatio-temporally^70,71^, and is critical to devise vector-control strategies based on the existing malaria vectors.

In Mizoram, ~ 54% of the rural households are engaged in shifting cultivation, also known as Jhum cultivation, which involves slashing and burning of selected forest area, followed by a short period of growing mixed crops, harvesting, following, and subsequently, the site will be abandoned^72,73^. In Mizoram, for Jhum cultivation, an estimated 0.2 million acre of forest land is burnt annually, resulting in significant greenhouse emissions^7^, which could be a contributor to the climate change trends. Furthermore, Jhum cultivation continuously exposes the farmers to malaria vectors as they stay and sleep in the forests. By opening up the forests, Jhum cultivation leads to associated streams, creating favorable breeding grounds for *An. minimus*^74^. In recent years, the Jhum cycle has reduced from 10–20 years to 2–3 years, and encroachment of virgin forested areas has led to new perennial malaria transmission zones^75^. As Jhum cultivation is widely practiced in Champhai and other districts bordering Myanmar (Fig. 7), there are possibilities for surge in malaria cases. In recent years, the sub-centers with > 1.0 API are increasing in the districts bordering Myanmar. In 2021, two sub-centers along the Myanmar border, where Jhum cultivation is widely practiced, recorded an API of > 100. Thus, malaria control strategies should continuously be implemented in this region and should track, test, and treat farmers involved in Jhum cultivation.

As our analysis shows, 5 of the 8 districts in Mizoram are high malaria transmission settings, and continuous exposure to *Plasmodium* in these endemic areas may result in low-density infections and/or asymptomatic carriers. The low-density infections from asymptomatic carriers can lead to malaria transmission^76,77^. For effective malaria control, strategies should include mass screening of the community with molecular tools. Continuous molecular surveillance and prompt treatment will greatly help lessen the malaria burden and break the transmission cycle in these high-transmission settings.

One of the important limitations is the non-availability of sub-center level data month-wise and year-wise for some of the districts. Also, absence of socio-economic status datasets at the sub-center level meant appropriate confounding factors were not included in the quasi-Poisson analysis. Even though we detail the role of conducive environmental factors, it will be hard to conclude malaria endemicity in the western districts is solely due to favorable climate unless confounding factors such as socioeconomic determinants are considered and controlled. It is also possible COVID-19 pandemic might have restricted the resources, which in turn could have displaced *Pv* hotspots in 2020 and 2021.

Overall, an integrated management approach involving regional cooperation, molecular surveillance in sentinel sites, laboratory investigation of ART resistance using RSA, community-level screening for detection of asymptomatic carriers, appropriate vector control strategies tailor-made for existing vectors, developing skilled manpower and infrastructure for molecular studies of malaria are required to effectively control malaria in this region.

## Supplementary Information

Supplementary Information 1.

Supplementary Information 2.

Supplementary Information 3.

Supplementary Information 4.

Supplementary Information 5.

Supplementary Information 6.

Supplementary Information 7.

Supplementary Information 8.

Supplementary Information 9.

Supplementary Information 10.

Supplementary Information 11.

Supplementary Information 12.

Supplementary Information 13.

Supplementary Information 14.

## Data Availability

All data analysed during this study are included in the manuscript (and the Supplementary Information files).
