# Supplementary material for: Malaria hotspots and climate change trends in the hyper-endemic malaria settings of Mizoram along the India–Bangladesh borders
Source: Sci Rep. 2023 Mar 20;13:4538. doi: 10.1038/s41598-023-31632-6 (PMC10025798; doi:10.1038/s41598-023-31632-6)
Supplement: Supplementary file 2 — Supplementary Information 2. [file 41598_2023_31632_MOESM2_ESM.docx]

| **Sl. No** | **Satellite image** | **Date of Acquisition** | **Bands used** | **Datum** | **UTM zone** |
| --- | --- | --- | --- | --- | --- |
| **1** | Landsat 8 | 06-03-2022 | 6 (Green, Red, Infrared, SWIR 1, SWIR 2, Cirrus) | WGS84 | 46 |
| **2** | Landsat 8 | 18-02-2022 | 6 (Green, Red, Infrared, SWIR 1, SWIR 2, Cirrus) | WGS84 | 46 |
| **3** | Landsat 8 | 18-02-2022 | 6 (Green, Red, Infrared, SWIR 1, SWIR 2, Cirrus) | WGS84 | 46 |
| **4** | Landsat 8 | 09-02-2022 | 6 (Green, Red, Infrared, SWIR 1, SWIR 2, Cirrus) | WGS84 | 46 |
| **5** | Landsat 8 | 08-01-2022 | 6 (Green, Red, Infrared, SWIR 1, SWIR 2, Cirrus) | WGS84 | 46 |
| **6** | Landsat 8 | 08-01-2022 | 6 (Green, Red, Infrared, SWIR 1, SWIR 2, Cirrus) | WGS84 | 46 |

**Table S2: Details of satellite images used for preparing LU/LC map of Mizoram (2022)**
