# Supplementary material for: Malaria hotspots and climate change trends in the hyper-endemic malaria settings of Mizoram along the India–Bangladesh borders
Source: Sci Rep. 2023 Mar 20;13:4538. doi: 10.1038/s41598-023-31632-6 (PMC10025798; doi:10.1038/s41598-023-31632-6)

# **Generalized Linear Regression**

Dependent Variable: Total Malaria Cases

**Summary of GLR Results [Model Type: Count (Poisson)]**

| **Variable** | **Coefficient**^a^ | **StdError** | **z-Statistic** | **Probability**^b^ | **VIF**^c^ |
| --- | --- | --- | --- | --- | --- |
| Intercept | -35.799000 | 1.869929 | -19.144572 | 0.000000^*^ | -------- |
| TEMP_MIN | 1.809538 | 0.037198 | 48.645549 | 0.000000^*^ | 76.473730 |
| TEMP_MAX | 0.432779 | 0.071096 | 6.087254 | 0.000000^*^ | 77.382394 |
| TEM_RANGE | -0.955982 | 0.051515 | -18.557363 | 0.000000^*^ | 41.019161 |
| RAIN | -0.008004 | 0.000197 | -40.624987 | 0.000000^*^ | 95.232156 |
| RH | 0.427702 | 0.010891 | 39.271252 | 0.000000^*^ | 20.347690 |
| ELEVATION | -0.003092 | 0.000029 | -106.130673 | 0.000000^*^ | 3.137949 |

**GLR Diagnostics**

|  |  |  |  |
| --- | --- | --- | --- |
| Input Features | Mala_SC_Para_Clean | Dependent Variable | SUM_TMC |
| Number of Observations | 385 | Akaike's Information Criterion (AICc)^d^ | 59681.000000 |
| Average Count | 107.197403 | Deviance Explained^e^ | 0.605028 |
| Joint Wald Statistic^f^ | 88972.886282 | Prob(>chi-squared), (6) degrees of freedom | 0.000000^*^ |

**Notes on Interpretation**

|  |  |
| --- | --- |
| * | An asterisk next to a number indicates a statistically significant p-value (p < 0.01). |
| a | Coefficient: Represents the strength and type of relationship between each explanatory variable and the dependent variable. |
| b | Probability: Asterisk (*) indicates a coefficient is statistically significant (p < 0.01) |
| c | Variance Inflation Factor (VIF): Large Variance Inflation Factor (VIF) values (> 7.5) indicate redundancy among explanatory variables. |
| d | Akaike's Information Criterion (AICc): Measures of model fit/performance. |
| e | % Deviance Explained: The proportion of dependent variable variance accounted for by the explanatory variable. |
| f | Wald Statistic: Asterisk (*) indicates overall model significance (p < 0.01). |

# **Generalized Linear Regression (after removing multicollinear parameters)**

Dependent Variable: Total Malaria Cases

##### Summary of GLR Results [Model Type: Count (Poisson)]

| **Variable** | **Coefficient**^a^ | **StdError** | **z-Statistic** | **Probability**^b^ | **VIF**^c^ |
| --- | --- | --- | --- | --- | --- |
| Intercept | 35.677106 | 0.559321 | 63.786432 | 0.000000^*^ | -------- |
| TEM_RANGE | -1.403859 | 0.015289 | -91.823169 | 0.000000^*^ | 6.421244 |
| RH | -0.087271 | 0.004914 | -17.758140 | 0.000000^*^ | 7.886764 |
| ELEVATION | -0.003578 | 0.000019 | -189.941043 | 0.000000^*^ | 1.687789 |

##### GLR Diagnostics

|  |  |  |  |
| --- | --- | --- | --- |
| Input Features | Mala_SC_Para_Clean | Dependent Variable | SUM_TMC |
| Number of Observations | 385 | Akaike's Information Criterion (AICc)^d^ | 65003.000000 |
| Average Count | 107.197403 | Deviance Explained^e^ | 0.568799 |
| Joint Wald Statistic^f^ | 83645.289908 | Prob(>chi-squared), (3) degrees of freedom | 0.000000^*^ |


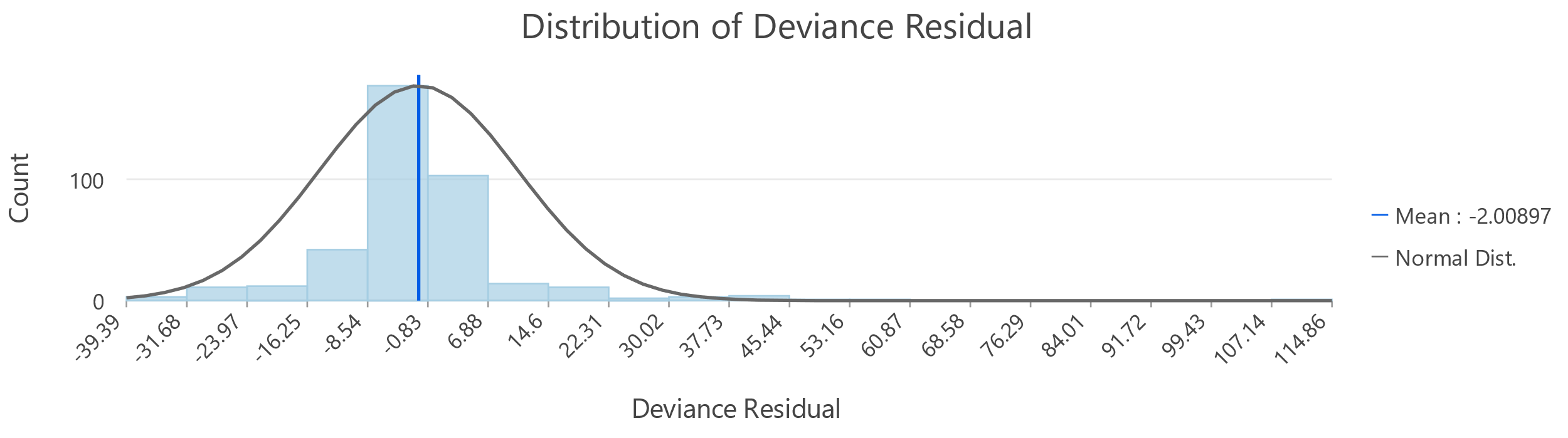

Supplement: Supplementary file 4 — Supplementary Information 4. [file 41598_2023_31632_MOESM4_ESM.docx]
