# Supplementary material for: Malaria hotspots and climate change trends in the hyper-endemic malaria settings of Mizoram along the India–Bangladesh borders
Source: Sci Rep. 2023 Mar 20;13:4538. doi: 10.1038/s41598-023-31632-6 (PMC10025798; doi:10.1038/s41598-023-31632-6)
Supplement: Supplementary file 5 — Supplementary Information 5. [file 41598_2023_31632_MOESM5_ESM.docx]

|  | **Temp. min** | **Temp. max** | **Temp. range** | **Rainfall** | **Relative humidity** | **Elevation** | **Built up/ Jhum** | **Dense forest** | **Mixed forest/shrub** |
| --- | --- | --- | --- | --- | --- | --- | --- | --- | --- |
| **Temp. min** | 1 |  |  |  |  |  |  |  |  |
| **Temp. max** | **0.83** | 1 |  |  |  |  |  |  |  |
| **Temp. range** | **-0.74** | -0.25 | 1 |  |  |  |  |  |  |
| **Rainfall** | **0.98** | **0.82** | **-0.75** | 1 |  |  |  |  |  |
| **Relative humidity** | **0.84** | 0.47 | **-0.91** | **0.88** | 1 |  |  |  |  |
| **Elevation** | **-0.80** | **-0.77** | 0.47 | **-0.79** | -0.61 | 1 |  |  |  |
| **Built up/ Jhum** | -0.06 | -0.15 | -0.07 | -0.07 | 0.01 | 0.13 | 1 |  |  |
| **Dense forest** | -0.19 | -0.18 | 0.11 | -0.18 | -0.14 | 0.18 | -0.28 | 1 |  |
| **Mixed forest/shrub** | 0.23 | 0.24 | -0.08 | 0.21 | 0.14 | -0.24 | -0.12 | **-0.92** | 1 |

**Supplementary Table S5: Correlation matrix between malaria cases and environmental variables**
