# Supplementary material for: Malaria hotspots and climate change trends in the hyper-endemic malaria settings of Mizoram along the India–Bangladesh borders
Source: Sci Rep. 2023 Mar 20;13:4538. doi: 10.1038/s41598-023-31632-6 (PMC10025798; doi:10.1038/s41598-023-31632-6)
Supplement: Supplementary file 6 — Supplementary Information 6. [file 41598_2023_31632_MOESM6_ESM.docx]

**Optimized Hot Spot Analysis (Spatial Statistics)**

**Summary**

- Given incident points, creates a map of statistically significant hot and cold spots using the Getis-Ord Gi* statistic. It evaluates the characteristics of the input feature class to produce optimal results.

**Initial Data Assessment**

- There are 385 valid input features.

Evaluating the Analysis Field values....

**AVG_API Properties:**

|  |  |
| --- | --- |
| Min | 0.0000 |
| Max | 137.5600 |
| Mean | 7.6941 |
| Std. Dev. | 16.4546 |

Looking for locational outliers....

- There were 2 outlier locations; these will not be used to compute the optimal fixed distance band.

**Scale of Analysis**

Looking for an optimal scale of analysis by assessing the intensity of clustering at increasing distances....

- The optimal fixed distance band is based on peak clustering found at 23457.8424 Meters
- Incident Data Aggregation Method is COUNT_INCIDENTS_WITHIN_FISHNET_POLYGONS

**Hot Spot Analysis**

Finding statistically significant clusters of high and low AVG_API values....

- There are 231 output features statistically significant based on an FDR correction for multiple testing and spatial dependence.
- .5% of features had less than 8 neighbors based on the distance band of 23457.8424 Meters
