# Supplementary material for: Malaria hotspots and climate change trends in the hyper-endemic malaria settings of Mizoram along the India–Bangladesh borders
Source: Sci Rep. 2023 Mar 20;13:4538. doi: 10.1038/s41598-023-31632-6 (PMC10025798; doi:10.1038/s41598-023-31632-6)
Supplement: Supplementary file 7 — Supplementary Information 7. [file 41598_2023_31632_MOESM7_ESM.docx]

**Table S7: Demographic (as per 2011 census) and geographic characteristics of districts in Mizoram.**

| District | Mamit | Kolasib | Aizawl | Champhai | Serchhip | Lunglei | Lawngtlai | Siaha |
| --- | --- | --- | --- | --- | --- | --- | --- | --- |
| Area (sq. km.) | 3,025 | 1,382 | 3,576 | 3,185 | 1,421 | 4,536 | 2,557 | 1,399 |
| Total Population | 86,364 | 83,955 | 4,00,309 | 1,25,745 | 64,937 | 1,61,428 | 1,17,894 | 56,574 |
| Population Density (sq. km ) | 29 | 61 | 112 | 39 | 46 | 36 | 46 | 40 |
| Rural (%) | 82.7 | 44.2 | 21.4 | 61.4 | 50.7 | 57.4 | 82.3 | 55.6 |
| Urban (%) | 17.3 | 55.8 | 78.6 | 38.6 | 49.3 | 42.6 | 17.7 | 44.4 |
| Sex Ratio (females/1000 males) | 924 | 956 | 1009 | 981 | 976 | 944 | 945 | 978 |
| Literacy (%) | 84.9 | 92.4 | 97.7 | 94.6 | 97.5 | 85.5 | 57.1 | 87.3 |
| Worker Population Rate (%) | 41.9 | 35.4 | 37.8 | 42.2 | 46.0 | 38.42 | 32.3 | 26.0 |
| Mean Temperature (maximum) (℃) | 35.2 | 35.7 | 34.4 | 33.9 | 34.2 | 35.2 | 35.6 | 34.9 |
| Mean Temperature (minimum) (℃) | 13.9 | 13.8 | 12.8 | 11.3 | 12.0 | 13.3 | 14.0 | 12.3 |
| Average Rainfall (mm) | 1,807 | 1,744 | 1,407 | 925 | 1,177 | 1,514 | 1,719 | 1,304 |
| Average Relative Humidity (%) | 74.2 | 72.8 | 71.9 | 68.6 | 70.3 | 71.2 | 71.2 | 69.0 |
| Mean Elevation (above MSL) | 375.4 (28, 1470) | 267.3  (26, 1157) | 660.2  (11, 1900) | 1064.8  (265, 2139) | 859.7  (243, 1869) | 505.2  (14, 1744) | 324.4  (31, 1449) | 810.1  (47, 2155) |
| Dominating LU/LC | Evergreen forest & shrub land | Evergreen forest | Shrub land & built-up | Evergreen forest | Evergreen forest & shrub land | Shrub land | Shrub land | Evergreen & deciduous forests |
