# Supplementary material for: Malaria hotspots and climate change trends in the hyper-endemic malaria settings of Mizoram along the India–Bangladesh borders
Source: Sci Rep. 2023 Mar 20;13:4538. doi: 10.1038/s41598-023-31632-6 (PMC10025798; doi:10.1038/s41598-023-31632-6)
Supplement: Supplementary file 8 — Supplementary Information 8. [file 41598_2023_31632_MOESM8_ESM.docx]

| **Table S8: Land use / land cover (2021-2022) distribution (in sq.km) of Mizoram districts** | | | | | |  |  |  |  |
| --- | --- | --- | --- | --- | --- | --- | --- | --- | --- |
|  |  |  |  |  |  |  |  |  |  |
| **Class \ District** | **Aizwal** | **Champhai** | **Kolasib** | **Lawngtlai** | **Lunglei** | **Mamit** | **Siaha** | **Serchhip** | **Mizoram (total)** |
|  |  |  |  |  |  |  |  |  |  |
| **Waterbody** | 8.82 (0.26) | 0.47 (0.01) | 31.27 (2.33) | 3.47 (0.18) | 6.64 (0.15) | 5.77 (0.19) | 1.40 (0.07) | 0.22 (0.02) | **58.07 (0.28)** |
| **Built-up** | 51.10 (1.49) | 49.03 (1.48) | 13.35 (1.00) | 12.34 (0.62) | 30.70 (0.68) | 10.65 (0.35) | 14.97 (0.78) | 15.28 (1.13) | **197.41 (0.95)** |
| **Dense Forest** | 1694.62 (49.48 | 1734.28 (52.23) | 368.82 (27.48) | 875.85 (44.25) | 1833.53 (40.73) | 904.35(29.73) | 902.63 (47.33) | 658.46 (48.66) | **8972.53 (42.99)** |
| **Mixed Forest** | 1199.59 (35.02) | 760.22 (22.90) | 501.39 (37.36) | 601.14 (30.37) | 1632.53 (36.27) | 1294.36 (42.55) | 486.39 (25.51) | 426.91 (31.55) | **6902.48 (33.07)** |
| **Shrub** | 395.96 (11.56) | 427.72 (11.88) | 406.42 (30.28) | 398.47 (20.13) | 888.51 (19.74) | 793.20 (26.08) | 437.69 (22.95) | 168.16 (12.43) | **3916.11 (18.76)** |
| **Cropland** | 5.20 (0.15) | 21.38 (0.64) | 10.09 (0.75) | 8.89 (0.45) | 11.20 (0.25) | 2.63 (0.09) | 9.75 (0.51) | 9.78 (0.72) | **78.92 (0.38)** |
| **Jhum Cultivation** | 69.64 (2.03) | 327.26 (9.86) | 10.77(0.80) | 79.29 (4.01) | 98.60 (2.19) | 30.79 (10.1) | 54.09 (2.84) | 74.26 (5.49) | **744.69 (3.57)** |
|  | **3424.90** | **3320.36** | **1342.10** | **1979.45** | **4501.69** | **3041.74** | **1907.00** | **1353.06** | **20870.297** |
|  |  |  |  |  |  |  |  |  |  |
|  |  |  |  |  |  |  |  |  |  |

*****percent proportion of LU/LC is given in brackets
