# Supplementary material for: Malaria hotspots and climate change trends in the hyper-endemic malaria settings of Mizoram along the India–Bangladesh borders
Source: Sci Rep. 2023 Mar 20;13:4538. doi: 10.1038/s41598-023-31632-6 (PMC10025798; doi:10.1038/s41598-023-31632-6)
Supplement: Supplementary file 9 — Supplementary Information 9. [file 41598_2023_31632_MOESM9_ESM.docx]

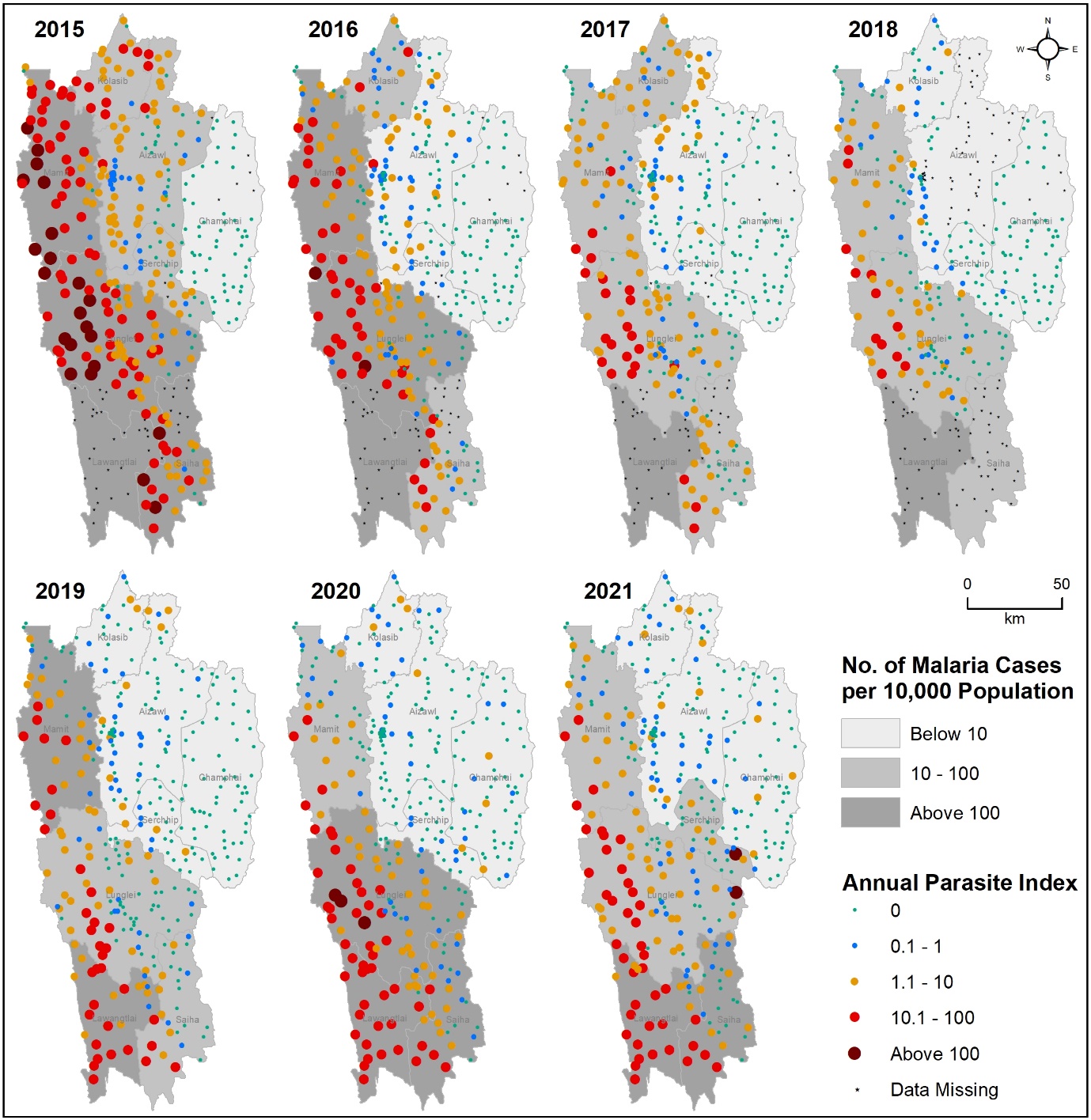


**Supplementary Figure S9.** Distribution of Annual Parasite Index (API) across 385 sub-centers of Mizoram from 2015 to 2021. The size (larger) and color (darker) of the dots show the distribution (higher) of API. The background grey shade represents district-wise normalised malaria cases where the darker shade shows the higher number of malaria cases per 10,000 population. Note that sub-center-wise data is not available for some of the years in Lawngtlai (2015-2018), Siaha (2018), and Aizawl East (2018) districts. The map was created using licensed version of ArcGIS 10.4 software by Esri. For more information about Esri® software, please visit [www.esri.com](http://www.esri.com).
