# Supplementary figures and images for: Malaria hotspots and climate change trends in the hyper-endemic malaria settings of Mizoram along the India–Bangladesh borders
Source: Sci Rep. 2023 Mar 20;13:4538. doi: 10.1038/s41598-023-31632-6 (PMC10025798; doi:10.1038/s41598-023-31632-6)

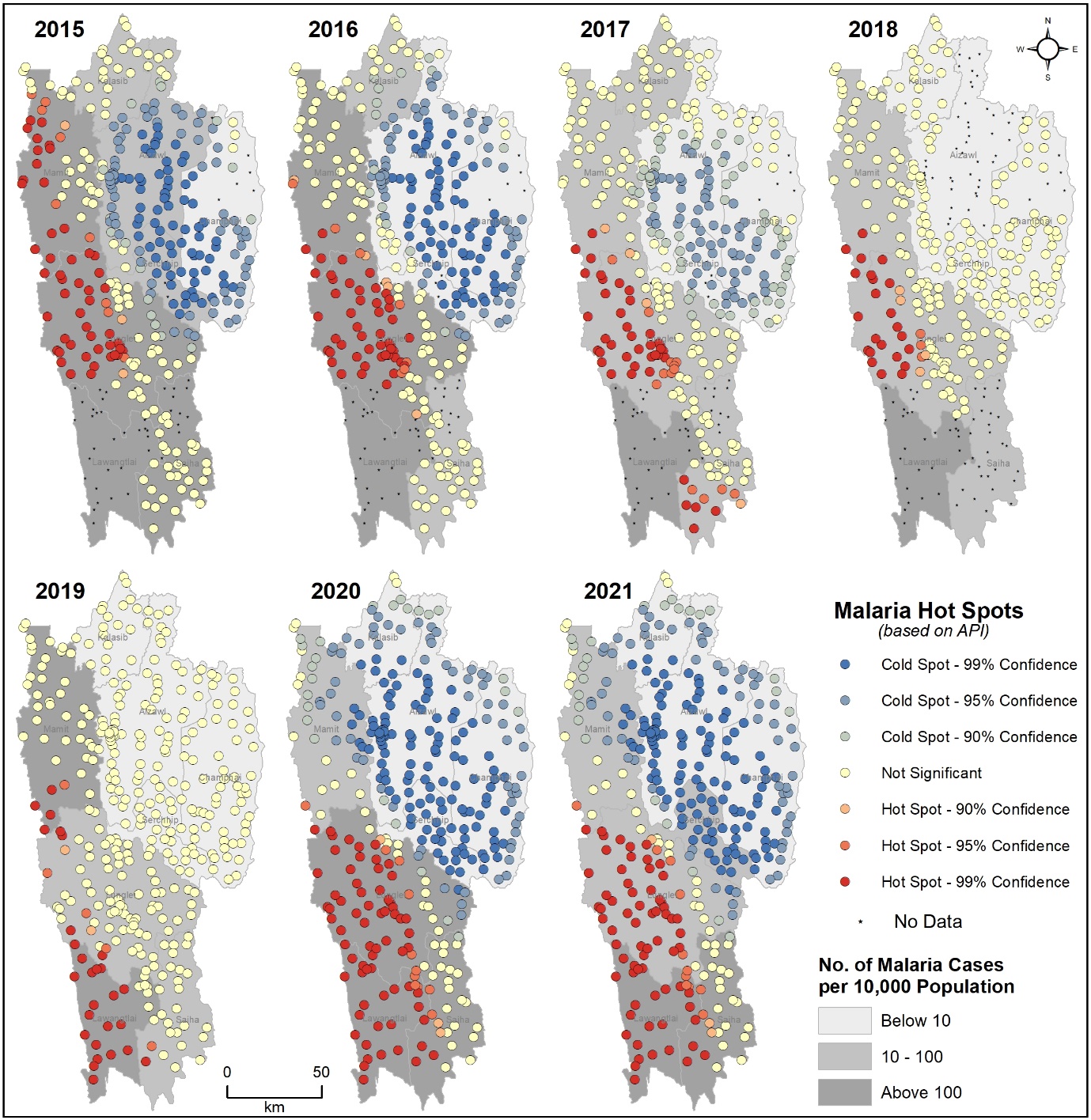


**Supplementary Figure S10.** Evolution of malaria hotspot over the years (2015-2021) in Mizoram.

Supplement: Supplementary file 10 — Supplementary Information 10. [file 41598_2023_31632_MOESM10_ESM.docx]

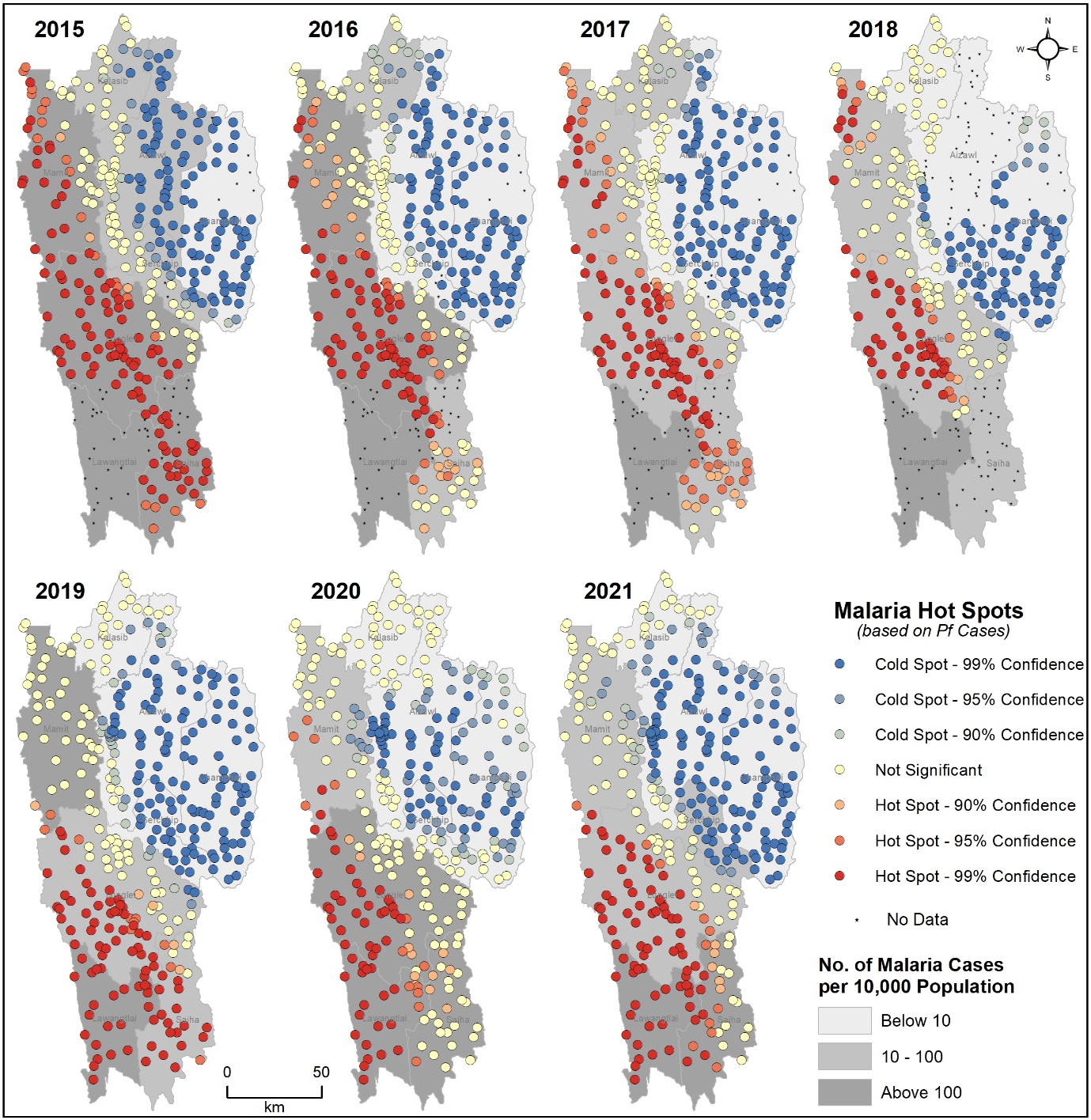


**Supplementary Figure S11.** Evolution of *Pf* malaria hotspot over the years (2015-2021) in Mizoram.

Supplement: Supplementary file 11 — Supplementary Information 11. [file 41598_2023_31632_MOESM11_ESM.docx]
