# Supplementary material for: Malaria hotspots and climate change trends in the hyper-endemic malaria settings of Mizoram along the India–Bangladesh borders
Source: Sci Rep. 2023 Mar 20;13:4538. doi: 10.1038/s41598-023-31632-6 (PMC10025798; doi:10.1038/s41598-023-31632-6)
Supplement: Supplementary file 12 — Supplementary Information 12. [file 41598_2023_31632_MOESM12_ESM.docx]

**Supplementary Table S12: Descriptive Statistics of LU/LC variables used in Kruskal Wallis test**

|  | **Malaria Cases** | | | |
| --- | --- | --- | --- | --- |
|  | **n** | **mean** | **SD** | **Median** |
| Built up/Jhum | 14 | 8.21 | 9.4 | 2 |
| Dense Forest | 261 | 69.25 | 228.98 | 26 |
| Mixed forest/Shrub | 110 | 209.76 | 594.2 | 139 |
