# Supplementary material for: Malaria hotspots and climate change trends in the hyper-endemic malaria settings of Mizoram along the India–Bangladesh borders
Source: Sci Rep. 2023 Mar 20;13:4538. doi: 10.1038/s41598-023-31632-6 (PMC10025798; doi:10.1038/s41598-023-31632-6)
Supplement: Supplementary file 13 — Supplementary Information 13. [file 41598_2023_31632_MOESM13_ESM.docx]

**
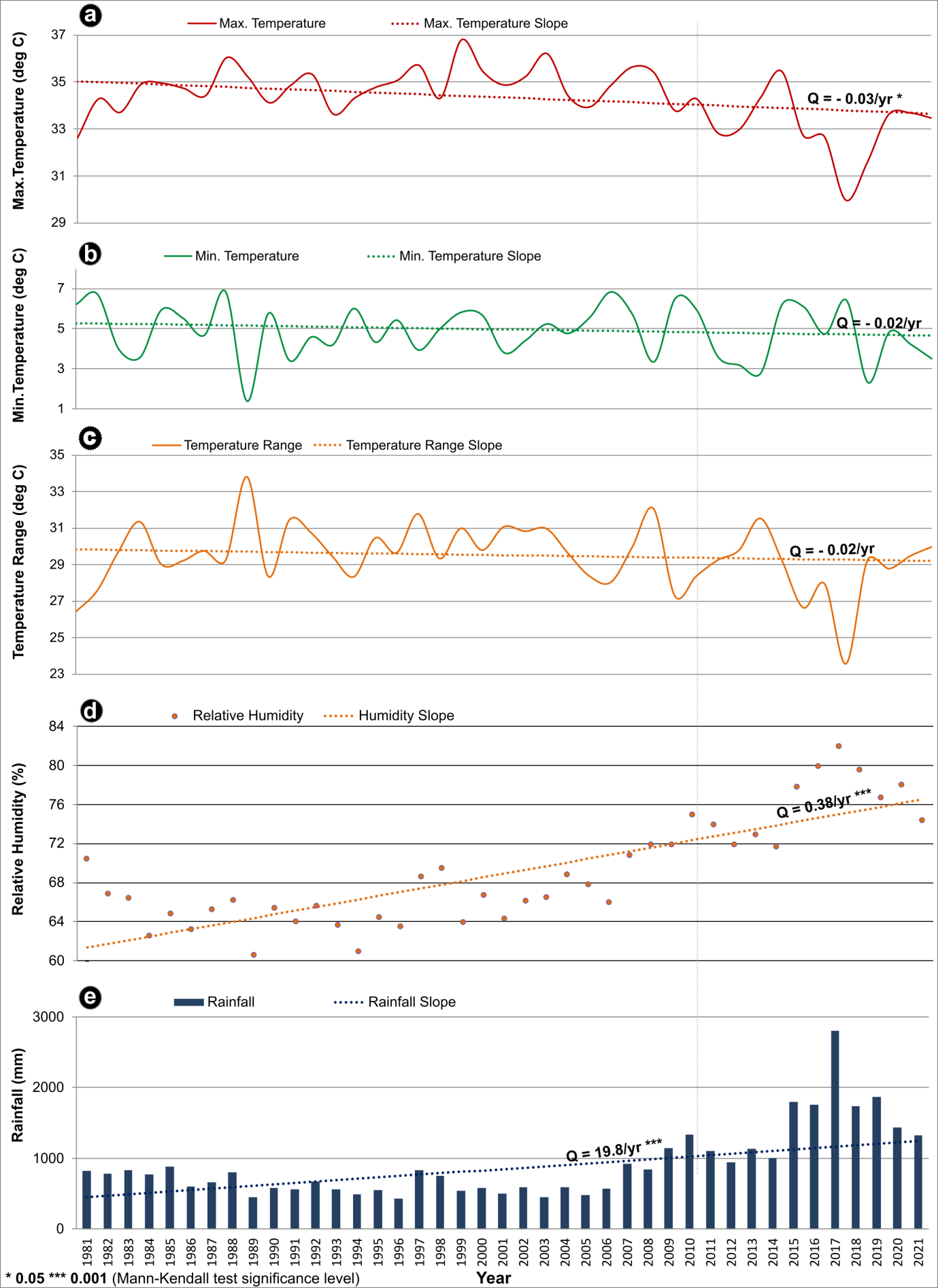
**

**Supplementary Figure S13.** Long-term trends in (a) Maximum temperature; (b) Minimum temperature; (c) Temperature range; (d) Relative humidity; (e) Rainfall in the Eastern regions of Mizoram. The dotted lines in the graphs show Sen’s slope, the rate of change per year (Q), and statistical significance (based on Z-value).
