# Supplementary material for: Malaria hotspots and climate change trends in the hyper-endemic malaria settings of Mizoram along the India–Bangladesh borders
Source: Sci Rep. 2023 Mar 20;13:4538. doi: 10.1038/s41598-023-31632-6 (PMC10025798; doi:10.1038/s41598-023-31632-6)
Supplement: Supplementary file 14 — Supplementary Information 14. [file 41598_2023_31632_MOESM14_ESM.docx]

**Supplementary Table S14. Trend statistics of climatic parameters (1981-2021) of Western and Eastern borders of Mizoram**

|  | **Mann-Kendall trend** | | | |  |  | **Sen's slope estimate** | | |  |  |  |  |
| --- | --- | --- | --- | --- | --- | --- | --- | --- | --- | --- | --- | --- | --- |
| **Location = Lawngtlai (Western Border)** | | | |  |  |  |  |  |  |  |  |  |  |
| **Time series** | **n** | **Test Z** | **Signific.** | **Q** | **Qmin99** | **Qmax99** | **Qmin95** | **Qmax95** | **B** | **Bmin99** | **Bmax99** | **Bmin95** | **Bmax95** |
| Temp. Max. | *41* | -4.77 | *** | -0.050 | -0.074 | -0.025 | -0.070 | -0.032 | 34.25 | 34.79 | 33.84 | 34.74 | 33.88 |
| Temp. Min. | *41* | 2.75 | ** | 0.015 | 0.001 | 0.028 | 0.005 | 0.025 | 16.76 | 17.03 | 16.49 | 16.96 | 16.51 |
| Temp. Range | *41* | -5.13 | *** | -0.065 | -0.092 | -0.039 | -0.085 | -0.045 | 17.44 | 18.01 | 16.88 | 17.86 | 17.04 |
| Relative Humidity | *41* | 6.29 | *** | 0.345 | 0.267 | 0.439 | 0.285 | 0.416 | 68.29 | 69.79 | 66.74 | 69.50 | 67.03 |
| Rainfall | *41* | 5.43 | *** | 34.277 | 20.596 | 48.70 | 24.45 | 45.351 | 1189.17 | 1429.60 | 916.44 | 1375.84 | 1008.31 |
|  |  |  |  |  |  |  |  |  |  |  |  |  |  |
| **Location = Champhai (Eastern Border)** | | | |  |  |  |  |  |  |  |  |  |  |
| **Time series** | **n** | **Test Z** | **Signific.** | **Q** | **Qmin99** | **Qmax99** | **Qmin95** | **Qmax95** | **B** | **Bmin99** | **Bmax99** | **Bmin95** | **Bmax95** |
| Temp. Max. | *41* | -2.04 | * | -0.034 | -0.079 | 0.009 | -0.069 | -0.001 | 35.03 | 35.90 | 34.28 | 35.76 | 34.42 |
| Temp. Min. | *41* | -0.76 |  | -0.016 | -0.068 | 0.038 | -0.054 | 0.024 | 5.28 | 6.20 | 4.33 | 6.14 | 4.52 |
| Temp. Range | *41* | -0.64 |  | -0.016 | -0.082 | 0.038 | -0.067 | 0.025 | 29.84 | 31.52 | 28.87 | 31.17 | 29.06 |
| Relative Humidity | *41* | 5.39 | *** | 0.379 | 0.238 | 0.514 | 0.270 | 0.482 | 61.35 | 63.86 | 57.99 | 63.58 | 58.87 |
| Rainfall | *41* | 3.40 | *** | 19.849 | 4.352 | 34.850 | 8.583 | 30.800 | 451.39 | 762.14 | 161.66 | 683.14 | 230.50 |
